# PAI-1-Dependent Endothelial Cell Death Determines Severity of Radiation-Induced Intestinal Injury

**DOI:** 10.1371/journal.pone.0035740

**Published:** 2012-04-26

**Authors:** Rym Abderrahmani, Agnes François, Valerie Buard, Georges Tarlet, Karl Blirando, Mohammad Hneino, Aurelie Vaurijoux, Marc Benderitter, Jean-Christophe Sabourin, Fabien Milliat

**Affiliations:** 1 Laboratory of Radiopathology and Experimental Therapeutics, Institute for Radiological Protection and Nuclear Safety, Fontenay-aux-Roses, France; 2 Laboratory of Biological Dosimetry, Institute for Radiological Protection and Nuclear Safety, Fontenay-aux-Roses, France; 3 Department of Pathology, Rouen University Hospital, Rouen, France; Institut Gustave Roussy, France

## Abstract

Normal tissue toxicity still remains a dose-limiting factor in clinical radiation therapy. Recently, plasminogen activator inhibitor type 1 (SERPINE1/PAI-1) was reported as an essential mediator of late radiation-induced intestinal injury. However, it is not clear whether PAI-1 plays a role in acute radiation-induced intestinal damage and we hypothesized that PAI-1 may play a role in the endothelium radiosensitivity. *In vivo*, in a model of radiation enteropathy in PAI-1 −/− mice, apoptosis of radiosensitive compartments, epithelial and microvascular endothelium was quantified. *In vitro*, the role of PAI-1 in the radiation-induced endothelial cells (ECs) death was investigated. The level of apoptotic ECs is lower in PAI-1 −/− compared with Wt mice after irradiation. This is associated with a conserved microvascular density and consequently with a better mucosal integrity in PAI-1 −/− mice. *In vitro*, irradiation rapidly stimulates PAI-1 expression in ECs and radiation sensitivity is increased in ECs that stably overexpress PAI-1, whereas PAI-1 knockdown increases EC survival after irradiation. Moreover, ECs prepared from PAI-1 −/− mice are more resistant to radiation-induced cell death than Wt ECs and this is associated with activation of the Akt pathway. This study demonstrates that PAI-1 plays a key role in radiation-induced EC death in the intestine and suggests that this contributes strongly to the progression of radiation-induced intestinal injury.

## Introduction

Radiation therapy is crucial in the therapeutic arsenal used for more than half of cancer patients. However, normal tissue side effects associated with this treatment limit the effective radiation dose than can be delivered to the tumor [1], [2]. Most patients treated with ionizing radiation will develop acute complications and 5 to 10% chronic late damage such as fibrosis. Initiation and progression of radiation-induced injury involves different mechanisms such as the coagulation system activation, DNA repair, cell death, inflammation, endothelium activation, angiogenesis and matrix remodeling [3].

The plasminogen activator (PA) system, which controls the formation and activity of plasmin, plays a key role in vascular homeostasis. PAI-1 (or standard gene symbol SERPINE1) belongs to the family of serine protease inhibitors and is the primary physiologic inhibitor of plasminogen activators in vivo [4]. Both plasminogen activators (u-PA and t-PA) convert plasminogen to plasmin, which degrades insoluble fibrin. PAI-1 inhibits u-PA and t-PA and thus reduces plasmin generation. PAI-1 is not only an antifibrinolytic molecule, but also plays a role in extracellular matrix remodeling by reducing plasmin-dependent matrix metalloproteinase (MMP) activation. The role of PAI-1 in fibrosis has been widely studied and it has been shown that PAI-1 is upregulated in several fibrotic diseases [5]–[8].

Radiation-induced endothelial injury has been described as a crucial event in initiation of normal tissue damage [1]. Radiation-induced activation of endothelial cells (ECs) is associated with a pro-inflammatory, pro-thrombotic and antifibrinolytic phenotype [9]. Precise regulation of the fibrinolytic system determines vascular homeostasis, but also physiological processes such as wound healing. *In vitro* radiation increases PAI-1 expression in cancer [10], [11] and normal cells [12]–[14]. Moreover, PAI-1 overexpression has been described in radiation-induced nephrosclerosis in rats [15], [16] and in human radiation enteritis [17]. Our group and others have described upregulation of PAI-1 in ECs *in vitro* and *in vivo* after irradiation [12], [18], [19], and radiation-induced intestinal damage in patients treated with radiotherapy is associated with upregulation of PAI-1 in the endothelium [12]. Recently, PAI-1 was demonstrated as playing a crucial role in radiation-induced intestinal fibrosis. In a model of radiation-induced enteropathy in mice, PAI-1 knockout mice are protected against intestinal radiation-induced injury with increased survival and better intestinal function compared with wild-type (Wt) mice [12]. However, the role of PAI-1 in radiation-induced acute side effects is still unclear. As described in our previous study, 40 to 45% of Wt mice died within 10 days after localized irradiation at 19 Gy, whereas no PAI-1 knockout mice died. The two survival curves separate within two days after irradiation, suggesting a contribution of PAI-1 in early events occurring after radiation exposure. Among acute effects observed in normal tissue response to high-dose radiation, depletion of microvascular and stem cell compartments is clearly determinant [20], [21]. Most studies show that gastrointestinal syndrome following total-body irradiation in mice is in part due to a destruction and sterilization of radiosensitive compartments such as stem/clonogenic epithelial cells and microvascular endothelium. PAI-1 has been described as playing either pro- or anti-apoptotic roles [22]–[24]. PAI-1 has an anti-apoptotic and neurotrophic action in the central nervous system [25], and is pro-angiogenic and anti-apoptotic in vascular tumor cells [26] and vascular smooth muscle cells [27], [28]. Paradoxically, primary ECs isolated from aortas of PAI-1 −/− mice are protected from wortmannin-induced apoptosis and have enhanced rates of proliferation [29], [30].

Here we hypothesized that PAI-1 may influence EC radiosensitivity and the aim of this work was to explore the effects of genetic deficiency on radiation-induced cell death of radiation-sensitive compartments of the intestine. We report a critical role of PAI-1 in radiation-induced microvascular EC apoptosis.

## Materials and Methods

### Mice and irradiation procedures

Experiments were performed on Wt C57BL/6J (PAI-1 +/+) and PAI-1 −/− mice (Charles River Laboratories) in compliance with legal regulations in France for animal experimentations. In total, 160 animals (10–12 weeks old) were included in this study. Animal care and experimental procedures were approved by the ethics committee of the Institute for Radiological Protection and Nuclear Safety (number T23, 05–09). Radiation-induced enteropathy was produced by exposure of a localized intestinal portion to a single ionizing radiation dose as previously described [12]. Briefly, mice were anesthetized with isoflurane and, after laparotomy, a 3-cm long intestinal segment (10 cm from the ileocecal valve) was exteriorized and exposed to a single dose of 19 Gy gamma irradiation (Co^60^ source, dose rate 1.2 Gy/min). Sham irradiation was performed by maintaining the intestinal segment exteriorized without radiation exposure. After radiation exposure or sham-irradiation, the exposed segment was returned to the abdominal cavity and peritoneum/abdominal muscles and skin were separately closed with interrupted sutures.

### Histology and immunohistochemistry

To perform global analyses of the irradiated tissues, histology and immunohistochemistry analyses were performed on different groups of animals. For routine histology analysis, intestines were fixed in 4% formaldehyde solution and embedded in paraffin. Longitudinal sections (5 µm) were stained with hematoxylin-eosin-saffron. Radiation injury was determined in a blinded manner independently by two authors using a described and validated radiation injury scoring system [12]. For immunohistochemistry experiments, intestinal tissues were embedded with Tissue-Tek OCT mounting media and frozen in isopentane cooled by liquid nitrogen. CD31/TUNEL and E-Cadherin/TUNEL double staining was performed on 5 µm frozen sections after fixation with 4% paraformaldehyde for 20 minutes. For CD31 immunostaining, sections were permeabilized with a PBS-0.1% Triton-0.1% sodium citrate solution for 2 minutes at 4°C and nonspecific sites were blocked in 3% Normal Goat Serum (Dako) diluted in PBS. Sections were then incubated with anti-CD31 antibody (clone 390, Abcam) 1∶50^e^ for 1 hour at room temperature. For E-cadherin immunostaining, sections were incubated in PBS-1% BSA-0.2% nonfat milk-0.3% Triton for 10 minutes and were incubated with anti-E-cadherin antibody (rat monoclonal ECCD-2, Zymed) at a dilution of 1∶200 for 1 hour at room temperature. Negative controls were not exposed to primary antibodies. All samples were incubated with an Alexa fluor 568-conjugated goat anti-rat antibody (Molecular Probes) 1∶200 for 1 hour. The first immunostaining was fixed with 4% paraformaldehyde for 10 minutes. TUNEL staining was performed using the *In situ* Cell Death Detection Kit (Roche Applied Science) according to the manufacturer's instructions. The ECs and apoptotic cells were counted in the lamina propria of 60 to 70 villi (full longitudinal sections of complete villi) from seven or eight different animals for each group. The apoptotic epithelial cells were counted in about 100 to 150 crypt sections per sample from the same animals.

Analyses of intestinal vascular density were performed after CD31/Sytox Green staining of 20 µm frozen sections. After fixation with 4% paraformaldehyde and permeabilization with TBS-0.15% Triton, sections were incubated with anti-CD31 antibody for 2 hours followed by incubation with Alexa fluor 568-conjugated goat anti-rat antibody. Nuclei were counterstained with Sytox Green (Invitrogen) according to the manufacturer's instructions. Confocal analyses were performed on a Bio-Rad MRC 1024 ES confocal imaging system. Z-stack images were collected at 1 µm steps. Images were imported and analyzed with Histolab software. Red fluorescence was automatically detected and its area was related to the villus area.

### Total RNA isolation, reverse transcription and real-time PCR

Total RNA was prepared with the total RNA isolation kit (Rneasy Mini Kit; Qiagen, Valencia, CA). Total RNA integrity was analyzed using Agilent 2100 and after quantification on a NanoDrop ND-1000 apparatus (NanoDrop Technologies, Rockland DE), reverse transcription was performed using the High Capacity Reverse Transcription Kit (Applied Biosystems) according to the manufacturer's instructions. Pre-developed TaqMan® Gene Expression Assays (Applied Biosystems) were used and PCR was performed with the ABI PRISM 7900 Sequence detection system (Applied Biosystems). PCR fluorescent signals were normalized to a PCR fluorescent signal obtained from the housekeeping gene GAPDH (for in vitro experiments) or 18S (for in vivo experiments). Relative mRNA quantification was performed by using the comparative ΔΔCT method.

### Cell culture and irradiation

Human umbilical vein endothelial cells (HUVECs) were obtained from Lonza and cultured in EGM-2 MV culture medium (Lonza) according to the manufacturer's instructions. Cells were used between passages 3 and 6 and were irradiated with a ^137^Cesium source. Levels of living cells after irradiation were determined by counting viable cells using a hemocytometer and the trypan blue exclusion method.

### Murine endothelial cells: preparation and characterization

Murine primary aortic ECs were isolated from Wt and PAI-1 −/− mice. Wt and PAI-1−/− mice (six mice/preparation) were anesthetized by intraperitoneal injection of a mixture of ketamine (100 mg.kg^−1^) and xylazine (10 mg.kg^−1^). The rib cage was cut and we identified the aortic arch, which is attached to the heart and links the kidney and iliac branch point. The aortic tree was then placed in PBS to remove debris and blood and transferred into complete medium EBM2-MV (Lonza) at room temperature. Aortas were cut into small sections (2 to 3 mm) and opened longitudinally. Each segment was positioned lumen side down onto culture dishes coated with Matrigel (Becton Dickinson) and placed in incubator at 37°C. After 3 days we observed some outgrowth from the tissue and culture medium was changed every 3 days At each change, one part of the Matrigel was removed, allowing the cells to adhere to the plastic. After twenty days, cells were trypsinized and labeled with CD105-PE antibody (e-biosciences d:1/25). Cells were sorted by flow cytometry using a BD Facsvantage TM apparatus. Sorted CD105-positive cells were directly recovered in complete medium. The purity of the EC population was checked after labeling with CD105-PE and CD106-FITC (e-biosciences) antibodies and flow cytometry analyses. More than 92- 96% of cells passed were CD105- and CD106-positive cells. Cells were routinely cultured in EBM2-MV and purity of ECs was checked until 12 passages. Before experiments using murine ECs from Wt and PAI-1 −/− mice, analyses of phenotype were completed by immunohistochemistry with CD105, CD106, CD31 and vWf labeling and mRNA detection with real-time PCR of EC markers: FLt-1, KDR,FLt-4, angiopoietin-1, angiopoeitin-2, Tie-2, VEGF-A, VEGF-B, CD105, CD106, CD54/ICAM-1, u-PA, tPA, uPAR and PAI-1.

### Tube formation assay

The ability of murine ECs to form capillary-like structures was studied. Briefly, 12-well culture plates were pre-coated with 650 µl of Matrigel. ECs obtained from Wt or PAI-1 −/− mice were plated at the same density and were irradiated 2 hours later at 10 or 20 Gy. 24 hours after irradiation, capillary like-structures were quantified. For each individual point 8 images in different view-fields were realized with size-calibrated fields of vision using a standard microscope interfaced with Histolab software. In each image branch points were counted and averaged.

### Establishment of PAI-1-expressing stable cell lines

Construction of pCMV6-Neo-PAI-1 plasmid: pCMV6-Neo plasmid and pCMV6-XL5-PAI-1 plasmid (both from Origene) were digested with NotI (Promega). Digested pCMV6-Neo plasmid was then dephosphorylated with TSAP (Promega). pCMV6-Neo and pCMV6-XL5-PAI-1 digestion products were each gel-purified (Promega Wizard SV Gel and PCR Clean-Up). The 538 kb dephosphorylated and NotI-digested pCMV6-Neo fragment was ligated with the 3.2 kb NotI-digested PAI-1 fragment using Quick T4 DNA Ligase (Biolabs). The reaction mixtures were purified with the Geneclean III kit (QBiogene) and transformed into chemocompetent E. coli strain XL10-Gold (Stratagene). Transformation mixtures were spread on LB agar plate with ampicillin and plasmid DNA was obtained from minicultures (Promega Wizard Plus SV Minipreps). Transformants were screened by ApaLI digestion and one positive clone was amplified by LB culture supplemented with 0.1 mg/ml ampicillin. Pure plasmid was obtained using Qiagen Endofree Plasmid Maxi Kit. pCMV6-Neo-PAI-1 plasmid DNA integrity was checked by agarose gel analysis after ApaLI digestion and confirmed by sequencing. HUVECs (passage 3) were transfected with pCMV6-Neo-PAI-1 plasmid using the Amaxa nucleofection method according to the manufacturer's instructions. Cells were cultured for 7 days in complete medium supplemented with 100 µg/mL of G418 (Invitrogen). Clones were obtained after three passages and culture complete medium supplemented with 50 µg/mL of G418. Overexpression of PAI-1 was confirmed by western blot for each clone compared with control HUVECs used at the same passage.

### Clonogenic assays

The radiosensitivity of HUVECs overexpressing PAI-1 was assayed by clonogenic assay. Briefly, cells were seeded in 6-well culture plates (1000 cells/well) and, three hours after plating, were irradiated at different doses (1, 2, 3 and 4 Gy). Twelve days after irradiation, cells were fixed and stained with an absolute methanol solution containing 0.25% crystal violet and 3% paraformaldehyde. Colonies containing more than 50 cells were counted and the surviving fraction was calculated according to the following formula: (Number of colony counts/Number of cells plated)×(plating efficiency), where plating efficiency was defined as (Number of colony counts)/(Number of cells plated for non-irradiated controls). The surviving fraction at 2 Gy and survival curves were generated for each clone and for control HUVECs by combining data from two experiments with each experiment performed in triplicate.

### RNA interference

siRNA targeting PAI-1, PTEN or PDK-1 were from Dharmacon (Thermo Scientific). Cells were transfected with 100 nM of siRNA using Dharmafect as transfection reagent. Two days after transfection, cells were lysed for RNA and protein extraction as previously described. Knockdown efficiency was measured by real-time PCR and/or western blot.

### Western blotting

Total proteins were extracted using RIPA buffer supplemented with phosphatase and protease inhibitors. Protein concentration was determined using BCA assay (Sigma Aldrich) and equal amounts of protein were resolved by SDS-PAGE. The following protein-specific primary antibodies were used: anti-PAI-1 (Novocastra Laboratories Ltd, Newcastle, UK), anti-GAPDH (Biodesign), anti phospho-Akt (ser473), anti-Akt, anti-Phospho PTEN (ser380), anti-PTEN , anti Phospho-PDK-1 (ser241), anti BCL2, anti-BCL-XL, anti phospho-p38 MAPK (Thr180/Tyr182), anti-phospho Erk1/2 (Thr202/Tyr204), anti-NF-κB p65 (Cell Signaling Technology).

### Statistical analyses

All data are presented as mean ± SEM. For in vivo experiments, differences between different groups were tested for statistical significance using ANOVA. The 2-tailed Student's *t* test was used for all other experiments. A p value <0.05 was considered statistically significant.

## Results

### PAI-1 genetic deficiency is associated with reduced acute and late radiation-induced intestinal injury

**Figure 1:**
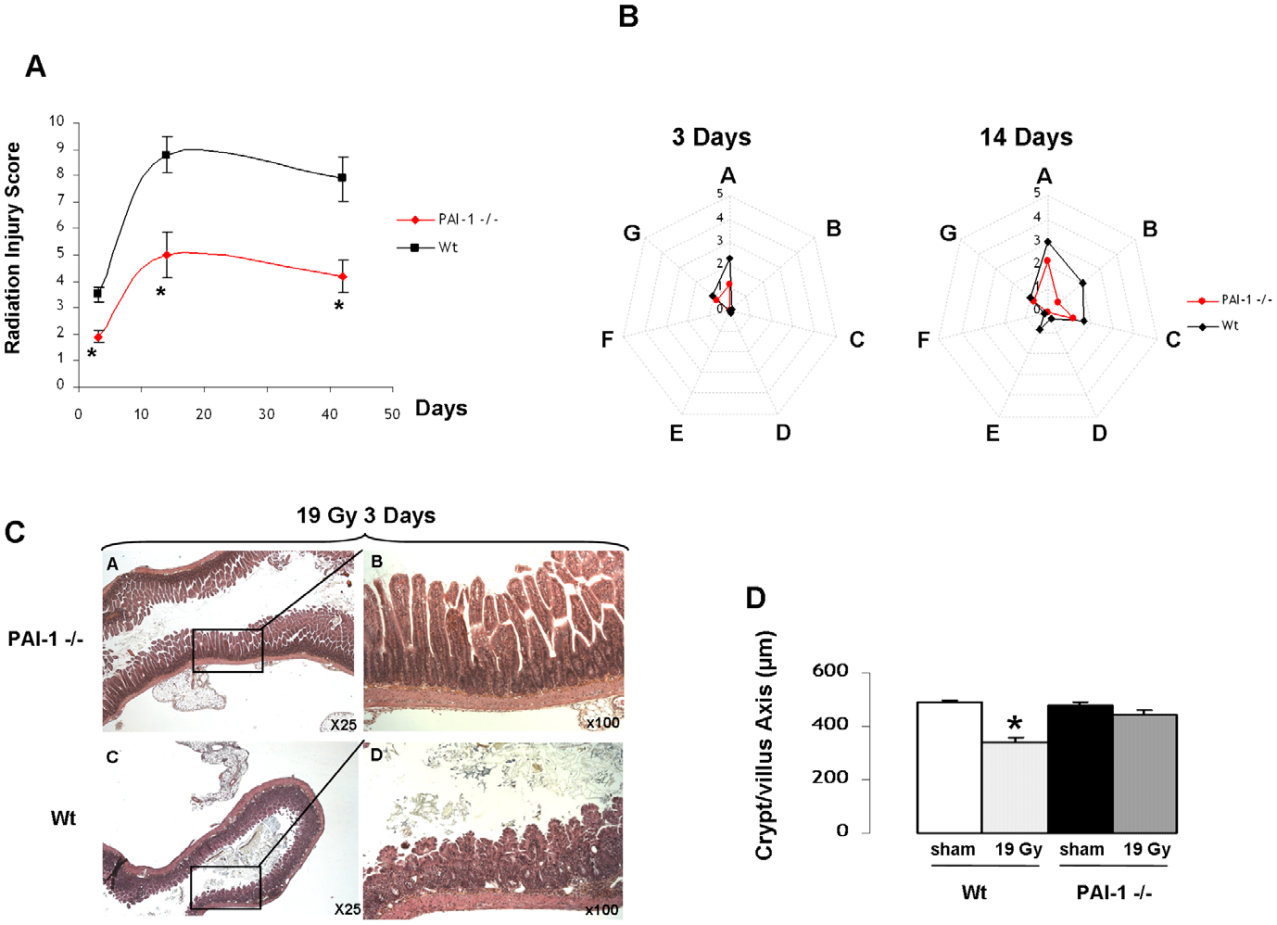
PAI-1 genetic deficiency is associated with reduced acute and late radiation-induced intestinal injury. Global radiation injury score (RIS) in Wt and PAI-1−/− mice 3, 14 and 42 days after irradiation (A). Detailed spider histograms of RIS 3 and 14 days after irradiation (n = 8 to 10 mice/group) (B). RIS includes A: mucosal ulceration, B: epithelial atypia, C: thickening of subserosa, D: vascular sclerosis, E: intestinal wall fibrosis, F: ileitis cystica profunda, G: lymph congestion. Representative images of intestinal damage 3 days after 19 Gy in Wt and PAI-1 −/− mice (C). Quantitative assessment of mucosal integrity in mice 3 days after 19 Gy (D). (n = 8 to 10 mice/group) # p<0.05 versus the three other groups.

In this study, we chose to use a model of localized irradiation of an intestinal segment. In this model, mRNA levels of components involved in the plasminogen activation system were determined by real-time PCR. Initiation of radiation-induced enteropathy in mice is associated with a strong upregulation of PAI-1 mRNA level and a slight upregulation of uPAR (Figure S1), with no modification of uPA and tPA. These results suggest that PAI-1 is the most probably part of the plasminogen activation system involved in radiation-induced enteropathy. Radiation injury scoring revealed that PAI-1 −/− mice are protected against radiation damage in both the late and acute phases, confirming previous results [12], [31] (Figure 1A). Detailed analyses performed at 3 and 14 days showed that differences in radiation injury between Wt and PAI-1 −/− mice are strongly associated with protection of the mucosae (Figure 1B–C). This was confirmed by the quantification of mucosal integrity 3 days after irradiation, showing that the length of the crypt/villus axis was reduced in Wt but not in PAI-1 −/− mice (Figure 1D).

### PAI-1 genetic deficiency is associated with reduced radiation-induced apoptosis *in vivo*

**Figure 2:**
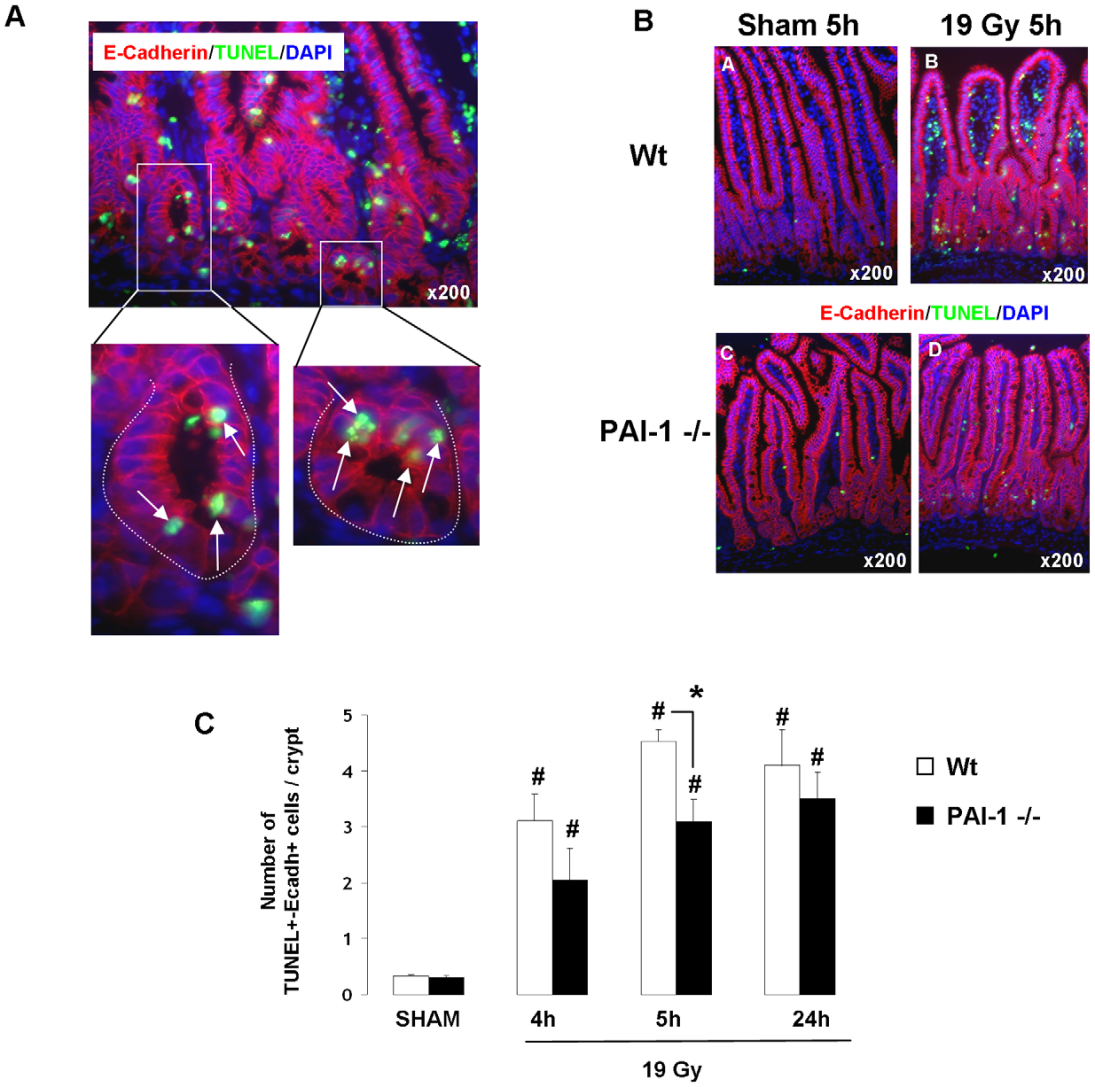
PAI-1 contributes slightly to radiation-induced intestinal epithelial cell apoptosis in crypts. High magnification of double TUNEL/E-cadherin staining in crypts in Wt mice 5 hours after 19 Gy. Arrows indicate apoptotic epithelial cells. Nuclei were counterstained with DAPI (blue) (A). Representative double TUNEL/E-cadherin staining in Sham (A–C) and irradiated (B–D) Wt (A–B) and PAI-1 −/− (C–D) mice 5 hours after 19 Gy. (B). Quantitative assessment of TUNEL+/E-cadherin+ cells in crypts in Wt and PAI-1 −/− mice 4, 5 and 24 hours after irradiation (C). Radiation-induced epithelial cell apoptosis in crypts was stimulated in both types of mice. The number of apoptotic epithelial cells was higher in Wt mice than in PAI-1 −/− mice only 5 hours after irradiation. (n = 6 mice/group) # p<0.05 versus sham mice with the same genotype. * p<0.05 between irradiated Wt and PAI-1 −/− mice.

**Figure 3:**
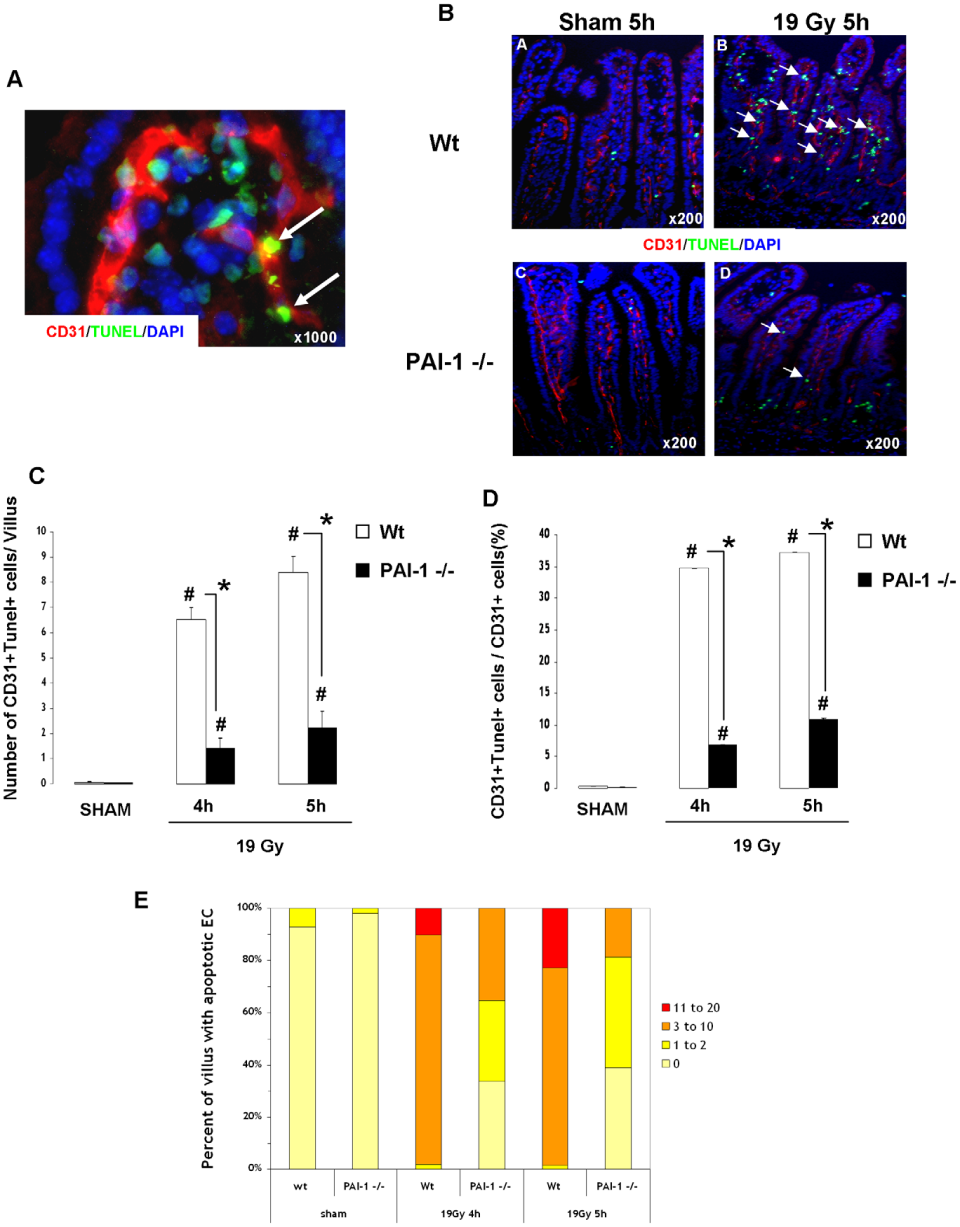
PAI-1 contributes strongly to radiation-induced intestinal endothelial apoptosis. High magnification of double TUNEL/CD31 staining in the villus lamina propria in Wt mice 4 hours after 19 Gy. Arrows indicate apoptotic endothelial cells. Nuclei were counterstained with DAPI (blue) (A). Representative double TUNEL/CD31 staining in Sham (A–C) and irradiated (B–D) Wt (A–B) and PAI-1 −/− (C–D) mice 5 hours after 19 Gy (B). Radiation-induced endothelial intestinal apoptosis was stronger in irradiated Wt mice than in irradiated PAI-1 −/− mice 4 hours after irradiation. Nuclei were counterstained with DAPI (blue). Quantitative assessment of TUNEL+/CD31+ cells in the villus lamina propria in Wt and PAI-1 −/− mice (C). Percentage of apoptotic endothelial cells/total endothelial cells in the villus lamina propria 4 and 5 hours after irradiation in Wt and PAI-1 −/− mice (D). Frequency of apoptotic endothelial cells in the villus lamina propria in Wt and PAI-1 −/− mice 4 and 5 hours after irradiation (E). (n = 6 mice/group) # p<0.05 versus sham mice with the same genotype. * p<0.05 between irradiated Wt and PAI-1 −/− mice.

**Figure 4:**
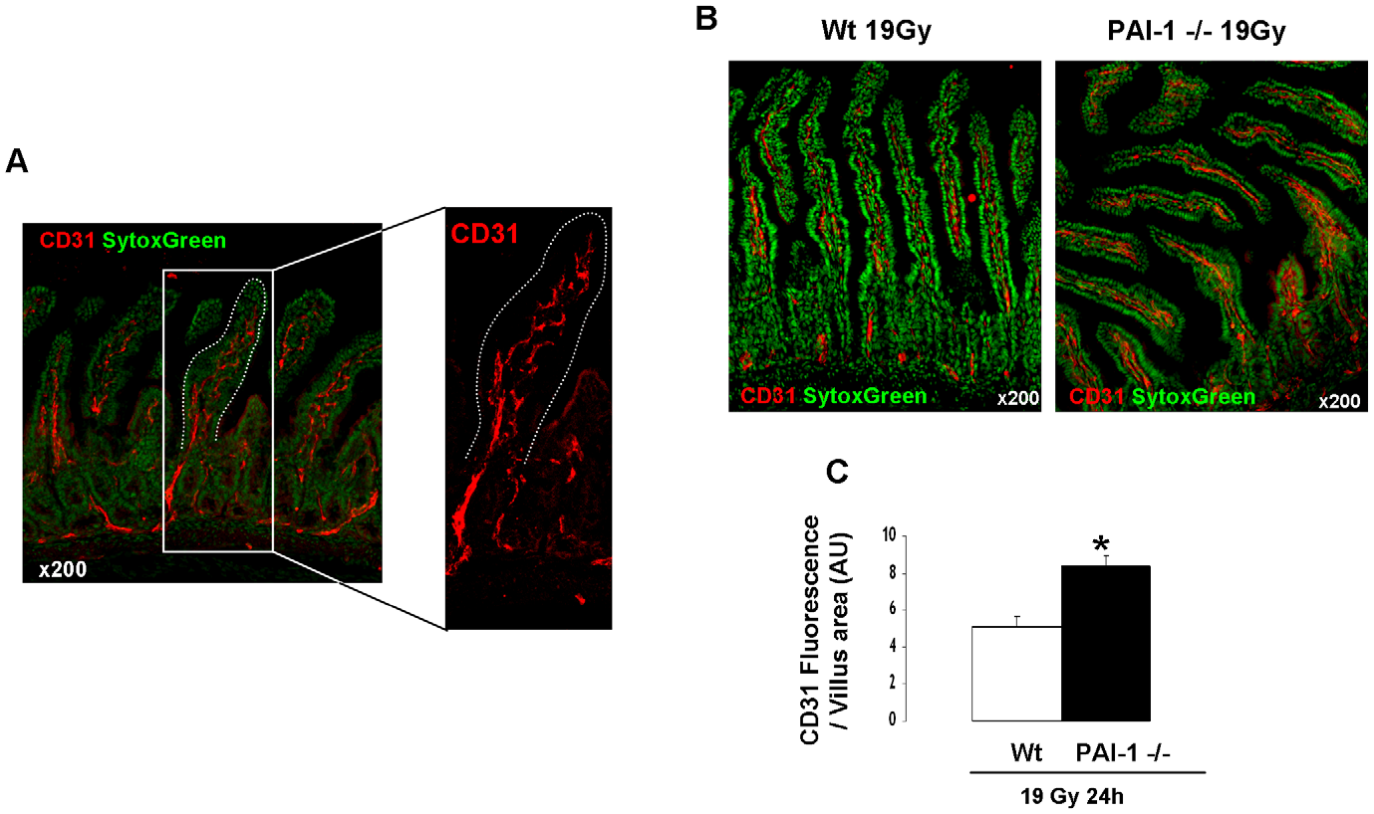
PAI-1 genetic deficiency is associated with reduced acute radiation-induced intestinal vascular injury. Intestinal vasculature was visualized by CD31 staining (red) and computerized with confocal microscopy imaging. Nuclei were counterstained with Sytox Green (A). Representative images of intestinal vasculature 24 hours after irradiation in Wt and PAI-1 −/− mice (B). Quantitative assessment of vasculature integrity 24 hours after 19 Gy (C). (n = 6 mice/group) # p<0.05.

Radiation-induced apoptosis is a crucial event in the acute effects of radiation. mRNA analyses determined in total intestinal tissues showed that genetic deficiency of PAI-1 is not associated with differences in mRNA levels of apoptosis/survival-related genes such as Bax, Caspase3, Bcl-2, Bcl-XL, Survivin, AKT1 and PTEN (Table S1). Moreover, irradiation increased Bax and Bcl-XL mRNA levels and decreased survivin mRNA levels, but no difference was observed between Wt and PAI-1 −/− mice (Figure S2). Irradiation had no effect on mRNA levels of caspase3, Bcl2, AKT1 and PTEN (Figure S2). These results obtained in total tissues suggest that differences between Wt and PAI-1 −/− mice in response to radiation injury are related to a specific compartment and not due to a global genetic-related effect. In order to evaluate the effect of PAI-1 deficiency in crypt epithelial cell death, E-cadherin/TUNEL double immunostaining was performed (Figure 2A). The number of apoptotic epithelial cells per crypt section increased 4, 5 and 24 hours after irradiation in both Wt and PAI-1 −/− mice, and no difference was found between Wt and PAI-1 −/− mice at 4 and 24 hours. We observed a slight decrease in the number of apoptotic epithelial cells per crypt section for irradiated PAI-1 −/− mice compared with irradiated Wt mice for the 5 hours time point (Figure 2B–C). These results suggest that another radiosensitive compartment may be involved. Single TUNEL staining showed that irradiation induced a significant increase in the number of TUNEL-positive cells in the villus lamina propria of Wt and PAI-1−/− mice with a 5.9-fold increase at 4 hours and a 9-fold increase at 5 hours in Wt mice, but only a 3.4-fold increase at 4 hours and a 4.7-fold increase at 5 hours in PAI-1−/− mice (Figure S3). In order to determine the level of EC apoptosis, we performed double staining of TUNEL and CD31 as a marker of ECs. Apoptotic ECs were identified by green nuclei surrounded by red CD31 staining (Figure 3A). Quantification of apoptotic ECs in the lamina propria of the villus showed an increase of apoptotic ECs 4 and 5 hours after radiation exposure in both strains, but the effect was stronger (p<0.05) in Wt mice compared with PAI-1 −/− mice (Figure 3B–C). The percentage of apoptotic ECs/total ECs revealed that there are 35% and 37% of ECs undergoing apoptosis respectively 4 and 5 hours after radiation exposure in Wt mice, whereas there are only 7% and 11% of apoptotic ECs in PAI-1−/− mice (Figure 3D). Moreover, PAI-1 genetic deficiency is associated with reduced microvascular EC damage, by decreasing the percentage of villi showing significant EC apoptosis (3 or more positive cells per villus) (Figure 3E). Microvascular network density after irradiation was assessed using CD31 immunostaining and computerized by confocal microscopy (Figure 4A). Staining areas of CD31 were compared between the two irradiated groups and the results showed greater microvascular density 24 hours after irradiation in PAI-1−/− mice compared with Wt mice (Figure 4B–C).

### PAI-1 −/− genetic deficiency in endothelial cells is associated with radiation resistance *in vitro*

**Figure 5:**
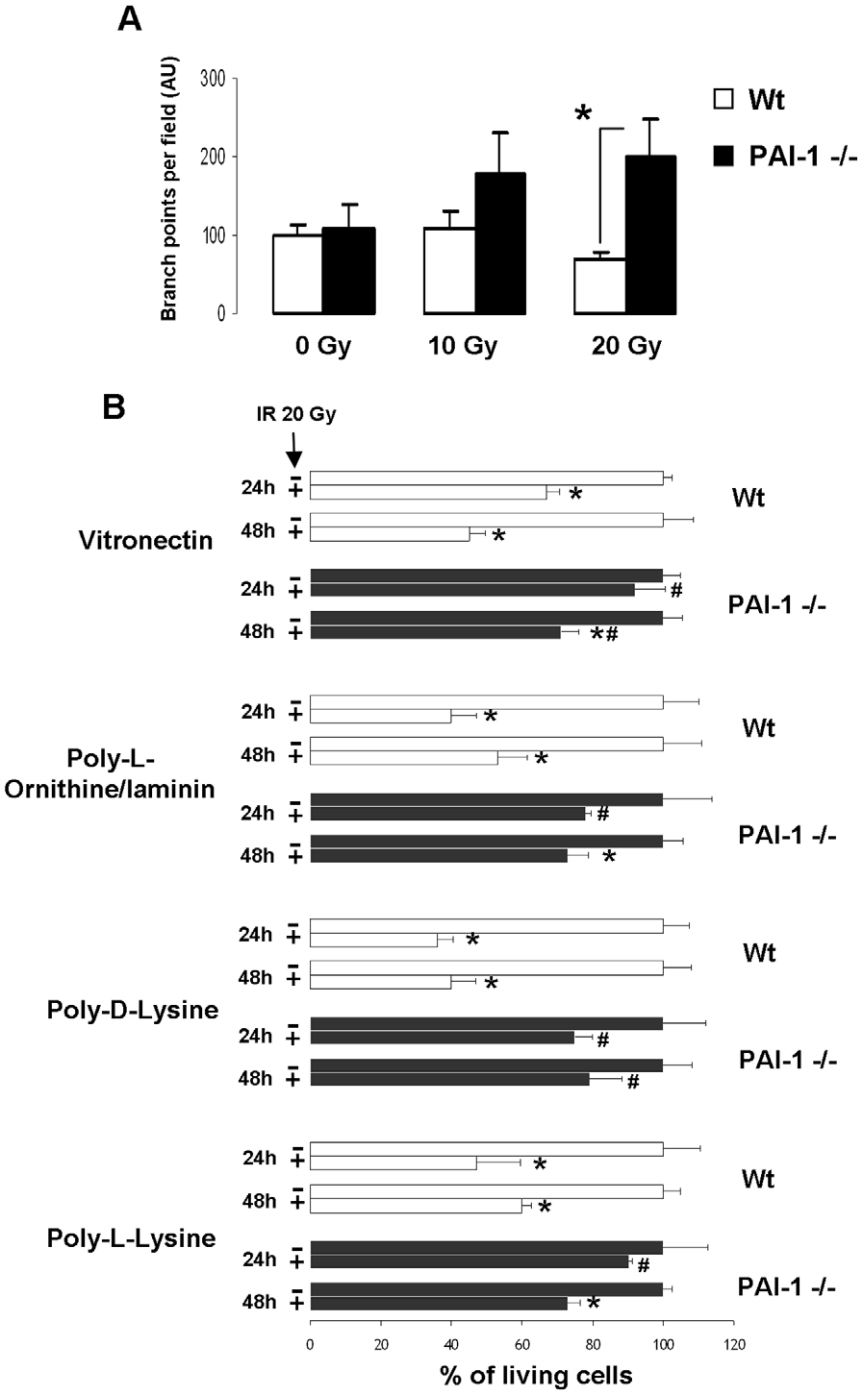
PAI-1 genetic deficiency in ECs is associated with increased survival after irradiation. In vitro Matrigel endothelial tube formation assay (A). Quantification was from three independent experiments performed in triplicate.* P<0.05. Percentage of living ECs 24 and 48 hours after 20 Gy. Wt and PAI-1 −/− ECs were plated on vitronectin, poly-L-ornithine/laminin, poly-D-lysine or poly-L-lysine (B). For each coating, results are the mean +/− SEM for two independent experiments performed in triplicate. * P<0.05 versus unirradiated cells for each genotype. # P<0.05 versus Wt irradiated cells.

ECs were prepared from Wt and PAI-1 −/− mice (Figure S4 *A–D*) and the endothelial phenotype was confirmed by flow cytometry, immunohistochemistry and detection of a panel of endothelial-specific transcripts by real-time PCR (Figure S4 *E–F*). Interestingly, irradiation increased PAI-1 expression in Wt ECs (Figure S4 *G*). In order to assess EC functionality after irradiation, Wt and PAI-1−/− cells were irradiated at 10 and 20 Gy in Matrigel. 24 hours after irradiation, PAI-1 −/− ECs were still able to form vascular-like networks, whereas irradiation of Wt ECs was associated with reduced ability to form vascular-like networks (p<0.05 between Wt and PAI-1−/− cells after 20 Gy; Figure 5A). To complete these results, ECs were plated on different coatings to increase cell adhesion. One and two days after 20 Gy irradiation, live cells were quantified and the results showed that PAI-1 −/− ECs were more resistant than Wt ECs (Figure 5B).

### Overexpression or knockdown of PAI-1 in endothelial cells influences sensitivity to ionizing radiation

**Figure 6:**
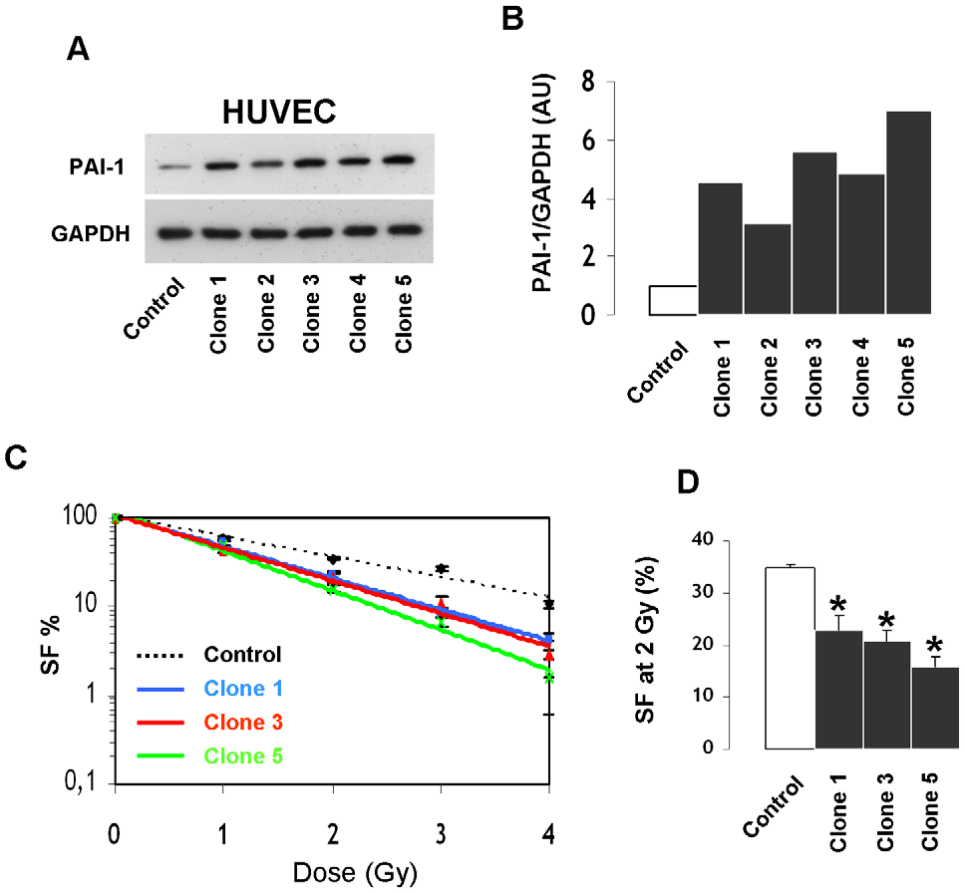
PAI-1 overexpression is associated with increased radiation sensitivity of endothelial cells. Representative western blot (A) and quantification of PAI-1 protein expression of five clones of HUVECs that stably overexpressed PAI-1 (B). Clonogenic assay in control HUVECs and clones 1, 3 and 5 (C). Surviving fraction after 2 Gy (D). Results are the mean +/− SEM (n = 6 per conditions) * p<0.05 versus control HUVECs.

**Figure 7:**
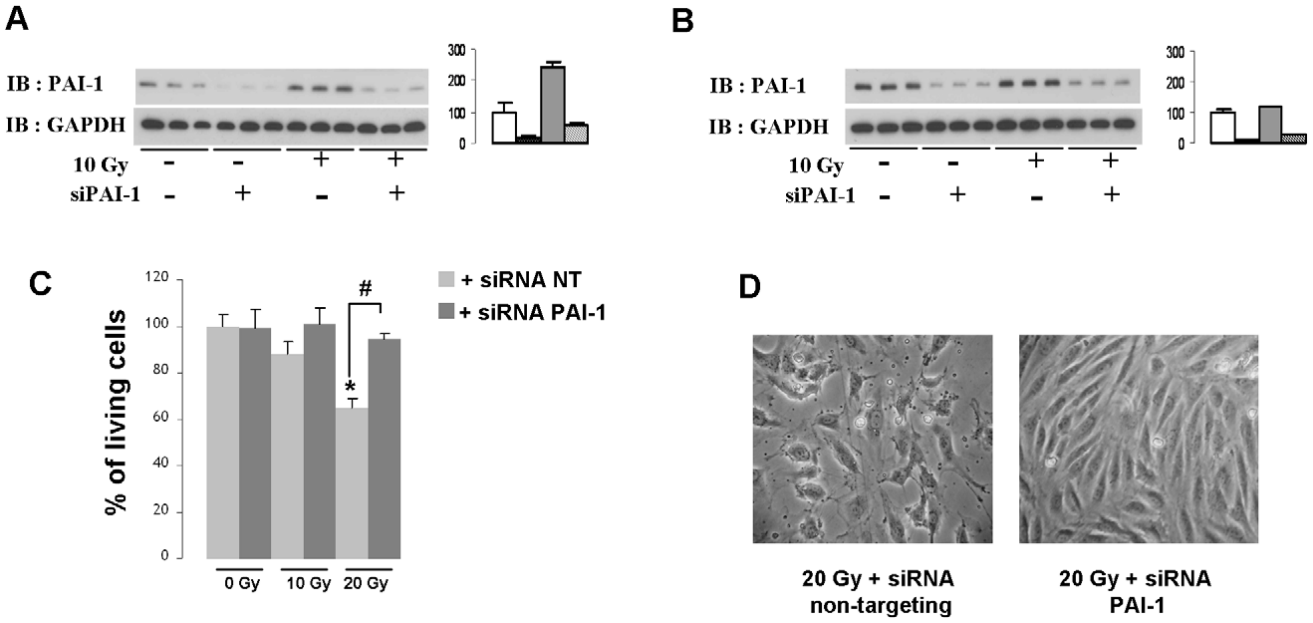
PAI-1 knockdown in human endothelial cells is associated with increased survival after irradiation. Representative western blots and quantification of PAI-1 protein expression 8 hours (A) and 24 hours (B) after irradiation in the absence or presence of siPAI-1. Percentage of living cells 24 hours after irradiation in the absence or presence of siPAI-1 (C). Results are the mean +/− SEM of two independent experiments performed in triplicate. * p<0.05 versus unirradiated HUVECs. Representative images of HUVECs 24 hours after 20 Gy in the absence or presence of siPAI-1 (D).

mRNA and protein levels of PAI-1 rapidly increased after irradiation in HUVECs (Figure S5). In order to know if PAI-1 plays a role in EC radiation sensitivity, stable HUVEC clones overexpressing PAI-1 protein at different levels were prepared (Figure 6*A–B*). Clonogenic assays were performed on 3 different clones and results were compared with those for control HUVECs. Results showed that overexpression of PAI-1 was associated with increased radiation sensitivity. Figure 6C shows that the surviving fraction for each clone decreased for irradiation doses from 0 to 4 Gy, and we show a reduced surviving fraction at 2 Gy in HUVECs overexpressing PAI-1 compared with control HUVECs (Figure 6D). Knockdown of PAI-1 in human ECs was performed using siRNA. Silencing efficiency was confirmed by real-time PCR and western blot (Fig S6). Western blot experiments showed that PAI-1 siRNA blunted the radiation-induced overexpression of PAI-1 (Figure 7A–B). Silencing of PAI-1 was associated with an increase in the percentage of living cells 24 hours after irradiation compared with non-targeting siRNA transfected cells (Figure 7C–D).

### PAI-1 influences the pro-survival Akt activation by a PTEN dependent and a PDK-1 independent mechanism

**Figure 8:**
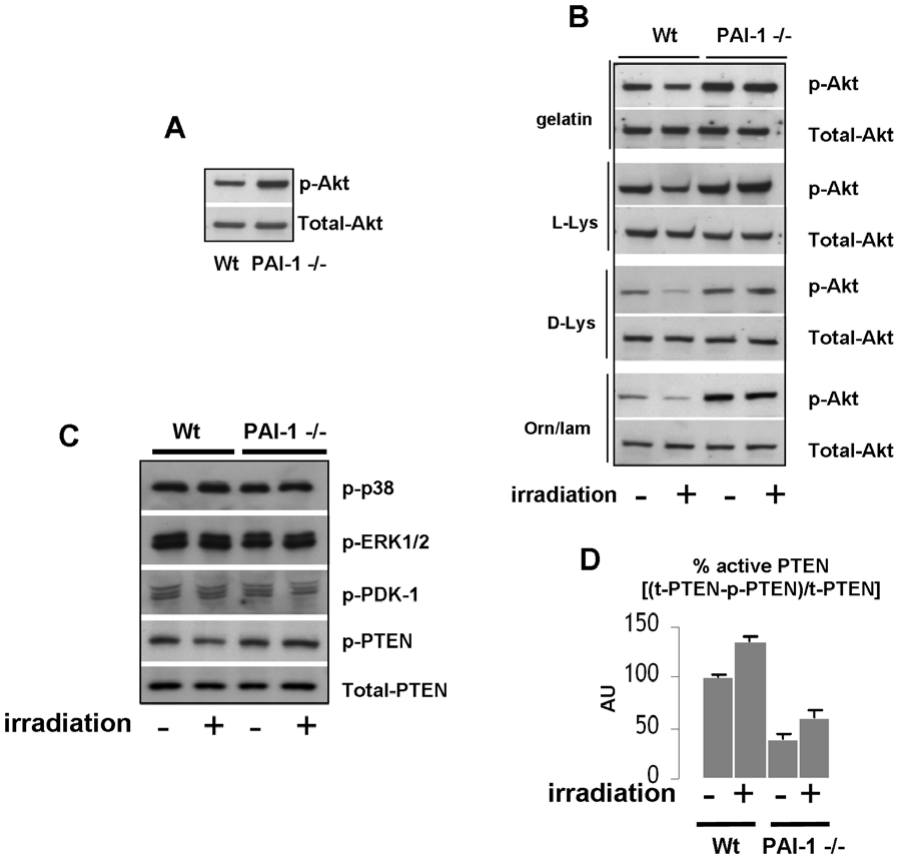
PAI-1 influences the pro-survival Akt pathway in murine endothelial cells. Representative western blots of phospho and total Akt in Wt and PAI-1 −/− ECs (A) and in Wt and PAI-1 −/− ECs platted on different coatings 24 hours after 20 Gy (B). Representative western blots of Phospho p38 MAPK, Phospho ERK1/2, Phospho PDK-1, Phospho PTEN and total PTEN in Wt and PAI-1 −/− ECs 24 hours after 20 Gy (C). Percentage of active PTEN in Wt and PAI-1 −/− ECs 24 hours after 20 Gy (D). All experiments were realized in triplicates.

**Figure 9:**
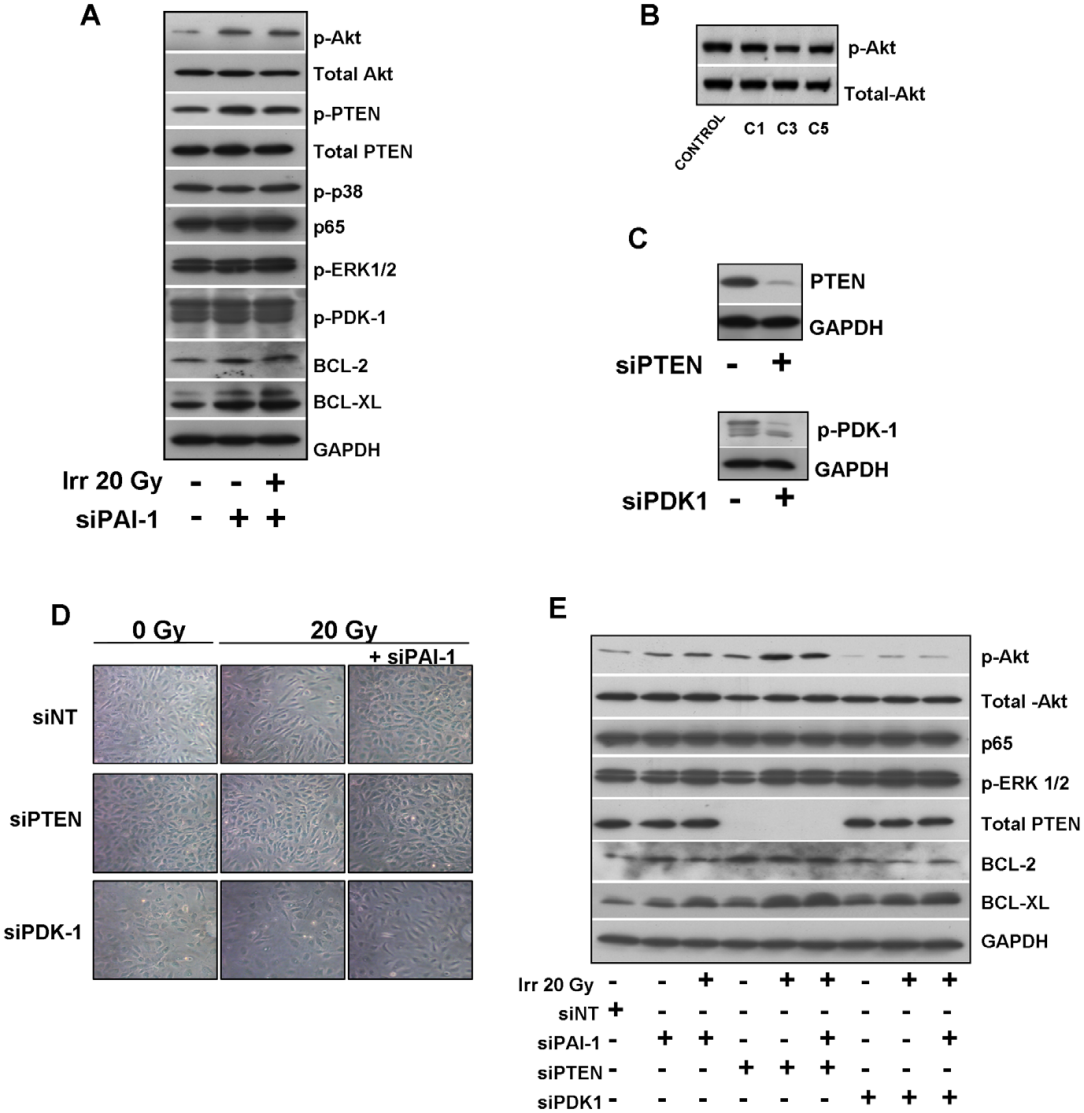
PAI-1 influences the pro-survival Akt pathway in human endothelial cells by PTEN inactivation. Representative western blots of phospho-Akt, total Akt, phospho-PTEN, total PTEN, phospho-p38MAPK, p65/RelA, phospho-ERK1/2, phospho-PDK-1, Bcl-2 and Bcl-XL in HUVECs transfected or not with siRNA PAI-1 24 hours after 20 Gy (A). Representative western blots of phospho and total Akt in HUVECs that stably overexpress PAI-1. Akt activation is reduced in human ECs that overexpress PAI-1 (B). Silencing efficiency determined by western blot in HUVECs transfected with siRNA PTEN or siRNA PDK-1 (C). Representative images of HUVECs knocked-down for PTEN, PDK-1, PAI-1, PTEN/PAI-1, or PDK-1/PAI-1 24 hours after 20 Gy (D). Representative western blots of phospho-Akt, total Akt, p65/RelA, phospho-ERK1/2, PTEN, BCL-2 and BCL-XL in HUVECs transfected with siRNA PTEN, PDK-1 and/or siRNA PAI-1 24 hours after 20 Gy(E) .

A Taqman low density apoptosis array approach was used to explore the effect of a human PAI-1 recombinant in 20 Gy irradiated HUVEC cells (Fig S7). Interestingly TLDA analysis reveals that levels of both pro-apoptotic and anti-apoptotic related genes are regulated in HUVECS exposed to 20 Gy with a strong increase of FAS and a strong decrease of the pro-survival gene BIRC5. No effect was observed following treatment with exogenous human PAI-1 recombinant compared with a latent form of PAI-1. These results suggest that PAI-1 does not influence radiation sensitivity of cells directly by an exocrine effect but probably by influencing intracellular signaling. So, we next explored consequences of PAI-1 deficiency or silencing on signaling pathways such as Akt, MAP kinase or NF-κb that can affect both pro-survival, pro- apoptotic or anti-apoptotic response of endothelial cells to radiation exposure. Whereas no differences were observed for the activation of p38 MAP Kinase, ERK 1/2, PAI-1 genetic deficiency is associated with activation of Akt (Figure 8A–C). After irradiation, the level of Phospho-Akt is decreased in Wt ECs whereas the high level of phospho-Akt in PAI-1 −/− ECs remains maintained (Figure 8B). Akt can be activated by phosphoinositide dependent kinase 1 (PDK1) and can be negatively regulated through the tumor suppressor phosphatase and tensin homologue deleted on chromosome ten (PTEN). Phospho-PDK1 levels remained unchanged whereas the inactivated form of PTEN (phosphorylated PTEN) is increased in PAI-1 −/− ECs compared with Wt ECs (Figure 8C). The percentage of active form of PTEN is decreased in PAI-1 −/− ECs (Figure 8D) suggesting that Akt activation associated with PAI-1 genetic deficiency is partly mediated by PTEN inactivation. In the same way, PAI-1 silencing in HUVECS is associated with increased activated Akt level (Figure 9A) whereas phospho-Akt expression is reduced in HUVEC that stably overexpress PAI-1 (Figure 9B). In siRNA PAI-1 transfected cells, no differences were observed for p38 MAP Kinase, ERK 1/2, p65 or phospho-PDK1 levels and the inactivated form of PTEN is increased (Figure 9A). Interestingly, PAI-1 genetic deficiency and PAI-1 silencing is also associated with increased levels of two anti-apoptotic proteins Bcl-2 and Bcl-XL. We evaluated consequences of PTEN and PDK1 silencing on Akt activation (Figure 9C). Whereas PDK-1 knockdown decreased the Phospho-Akt level, PTEN or PAI-1 knockdown protects cells from ionizing radiation and this is associated with Akt activation (Figure 9D–E). Phospho-Akt, Bcl-2 and Bcl-XL increase in cells transfected with siRNA PTEN, siRNA PAI-1 or both. This observation was confirmed in murine ECs (Fig S8) and suggest that pro-survival and anti-apoptotic pathways were involved in the PAI-1-dependent ionizing radiation response of endothelial cells.

## Discussion

Radiation-induced intestinal side effects remain a problem in pelvic cancer treatment because of a lack of therapeutics to prevent and/or reduce damage [32]. Most experiments in models of radiation-induced intestinal damage were performed after total-body irradiation with or without bone marrow transplantation. In these models, endothelial and epithelial cell apoptosis is widely described to be crucial in the initiation of gastrointestinal syndrome. It has been shown that EC apoptosis is the primary event initiating gastrointestinal syndrome in mice [20]. EC apoptosis occurs in the 4–6 hours following irradiation and precedes crypt epithelial clonogenic cell death. More recently, Rotolo *et al.* showed that bax−/− and bak −/− mice were rescued from gastrointestinal syndrome and demonstrated that bax and bak have nonredundant functional roles in radiation-induced microvascular cell death, but not in crypt stem cell death [33]. However, the relative contribution of microvascular endothelium and clonogenic crypt cells to radiation-induced gastrointestinal syndrome is still a matter of debate. This controversy is born from the prevailing concept established for many years claiming that gastrointestinal syndrome is mainly due to the crypt stem and clonogenic cell death [21]. After total-body irradiation, numerous studies have shown that gastrointestinal syndrome is associated with EC death [20], [34]–[37]. However, therapeutic strategies to rescue mice from gastrointestinal syndrome or transgenic mice protected from gastrointestinal syndrome are sometimes associated with endothelial protection [20], [36], [37], epithelial protection [34], [38] or both [35]. Finally, one group described no EC apoptosis in the intestine after specific irradiation of the endothelium with intravascular boronated liposomes [39], [40]. However, technical difficulties and artifacts in immunohistochemistry detection of apoptotic cells on paraffin sections could in part explain the controversy [41], [42]. Nevertheless, a lot of supportive evidence argues for a strong contribution of microvascular destruction in early intestinal radiation toxicity, but the causal relationship between EC apoptosis, crypt cell apoptosis and consequent progression of intestinal damage is still unclear.

Here we used a pertinent model of localized intestinal injury adapted to the study of acute and late effects [12], [43]. In this model we recently showed that PAI-1 pharmacological inhibition conferred temporary protection against early lethality and that PAI-1 genetic deficiency conferred complete protection [31]. These results suggested that PAI-1 could be involved in radiosensitivity of the microvascular and stem cell compartments. In the present work, immunohistochemical detection of apoptotic endothelial and epithelial cells was performed on frozen sections to avoid technique-dependent misinterpretation. In the model of radiation enteropathy, our results strongly support the concept that endothelial apoptosis is a key event involved in acute radiation intestinal injury and consequently in the progression of radiation-induced fibrosis. Reduced radiation-induced intestinal damage observed in PAI-1 −/− mice is associated with a strong reduction in EC death. This observation *in vivo* was confirmed *in vitro* with multiple approaches. First we show that, after irradiation, the functionality of murine ECs isolated from PAI-1−/− mice aorta is preserved. Using a functional test that integrates different parameters such as the adhesion rate, the ability to proliferate and to migrate, and the radiation-induced cell death test, we showed that PAI-1 −/− ECs are still able to form vascular-like tubes after irradiation. It has been shown that murine PAI-1−/− ECs have enhanced rates of proliferation compared with Wt ECs and are more resistant to wortmannin-induced apoptosis [29], [30]. Our results obtained in murine ECs plated on different surfaces coated with vitronectin, poly-L-ornithine/laminin, poly-L-lysine or poly-D-lysine show that PAI-1 −/− ECs are more resistant to radiation-induced cell death than Wt ECs. Moreover we provide evidence that PAI-1 genetic deficiency directly influences survival of ECs suggesting that the differences in abilities of Wt and PAI 1 −/− ECs to form vascular-like networks can be due to both lower capacity of Wt cells to proliferate and higher levels of radiation-induced apoptosis. To complete these observations, we modulated PAI-1 gene expression in HUVECs. With a classic test of radiosensitivity, we showed that overexpression of PAI-1 confers enhanced radiation sensitivity on the cells, and the surviving fraction is linked to the level of PAI-1 expression. In contrast, PAI-1 knockdown is associated with an increased rate of survival in HUVECs. Taken together, our results demonstrate that PAI-1 plays a key role in radiation-induced EC death and suggest that this contributes strongly to the progression of radiation-induced intestinal injury. PAI-1-dependent mechanisms associated with pro-apoptosis or pro-survival pathways in irradiated endothelium are unknown and require further study.

The role of PAI-1 in angiogenesis and apoptosis of vascular cells is controversial. PAI-1 is described as pro-angiogenic [44] or anti-angiogenic [45], [46]. For example, studies on prostatic tumor PC-3 cells conditionally expressing active PAI-1 regulated by doxycycline, showed that PAI-1 strongly decreased tumor progression through the destruction of tumor vascularization. The doxycycline-induced PAI-1 pool anti-angiogenic effect is due to an early wave of apoptosis in tumor ECs and this effect was described as vitronectin-dependent [47]. Clearly, the angiogenic effects of PAI-1 are dose-dependent [48]. At physiological concentration PAI-1 promotes angiogenesis whereas high concentrations of PAI-1 are anti-angiogenic. Interestingly, *in vitro*, the ceramide antagonist sphingosine-1-phosphate (S1P) protects endothelial cells from radiation-induced apoptosis [49] and, *in vivo*, S1P specifically protects intestinal microvascular endothelial cells through a mechanism involving activation of Akt [50]. Here, we showed that PAI-1 genetic deficiency is associated with Akt activation in endothelial cells and resistance to radiation-induced cell death. Akt activation can occurs by different mechanisms including negative regulation by PTEN or activation by the phosphoinositide-dependent protein kinase 1 (PDK1). In our experimental conditions, the PAI-1-dependent activation of Akt is not linked to PDK-1 activation. Phosphorylation of PTEN results in its inactivation and we report that PAI-1 genetic deficiency of murine ECs or knockdown of PAI-1 in HUVECs increase the inactivated form of PTEN. Consequently decreased active PTEN pool may probably lead to Akt activation. This is in accordance with Balsara et al who showed that PAI-1-deficient ECs have enhanced Akt(Ser(P)^473^) level due to enhanced inactivation of PTEN. This Akt hyperactivation is associated with increased levels of inactive caspase-9 and lower levels of active caspase-3, thus rendering PAI-1 −/− ECs resistant to spontaneous apoptosis or chemical-induced pro-apoptotic signals [29]. In addition, we observed that PAI-1 genetic deficiency or silencing is associated with increased expression of Bcl-2 and Bcl-XL. Interestingly Akt was reported to increase these two anti-apoptotic genes in endothelial cells contributing to cell survival [51], [52]. Because neither p38MAPk, ERK 1/2 or NF-κB seems to be influenced by PAI-1 knockdown, our results strongly suggest that the PAI-1 dependent Akt activation is involved in the overexpression of Bcl-2 and Bcl-XL. The precise mechanism by which PAI-1 influences survival pathways are still unclear and future researches are needed to answer this question.

In conclusion, our results demonstrate that PAI-1 genetic deficiency is associated with a strong anti-apoptotic effect on ECs in the acute phase, with higher microvascular density and decreased radiation-injury score during acute, sub-acute and late phases of radiation enteropathy. This study indicates that radiation-induced overexpression of PAI-1 in ECs has pro-apoptotic effects and contributes to the destruction of the microcirculation in the intestine. Further experiments are necessary to explore the molecular mechanisms involved and the sequential involvement of PAI-1 in the initiation and progression of radiation-induced fibrosis.

## Supporting Information

Figure S1 **Effect of irradiation on mRNA levels of PAI-1, uPAR, uPA and tPA in Wt and PAI-1 −/− mice.** mRNA levels of plasminogen activation system in Wt and PAI-1 −/− mice in total intestinal tissues were measured by Real time PCR 5 h and 24 h after irradiation. Results are +/− SEM (n = 6 mice/group). ND : not detected.
(TIF)

Figure S2 **Effect of irradiation on mRNA levels of Bax, Bcl-2, Caspase 3, Survivin, Bcl-XL, Akt and PTEN in Wt and PAI-1 −/− mice.** mRNA levels in Wt and PAI-1 −/− mice in total intestinal tissues were measured by Real time PCR 5 h and 24 h after irradiation. Results are +/− SEM (n = 6 mice/group).
(TIF)

Figure S3 **PAI-1 genetic deficiency is associated with reduced radiation-induced intestinal apoptosis.** Three cm of intestine from Wt and PAI-1 −/− mice was irradiated with a localized 19 Gy single dose. Apoptosis in irradiated intestine was assessed by TUNEL staining. (A) Representative TUNEL staining (green) in Wt (A) and PAI-1 −/− (B) mice 4 hours after irradiation. Nuclei were counterstained with DAPI (blue). (B) Quantitative assessment of TUNEL+ cells in the villus in Wt and PAI-1 −/− mice 4 and 5 hours after irradiation. (n = 6 mice/group) # p<0.05 versus sham mice with the same genotype. # p<0.05 between irradiated Wt and PAI-1 −/− mice.
(TIF)

Figure S4 **Preparation and characterization of mice endothelial cells isolated from aortas.** The aorta was harvested cut into pieces, opened longitudinally, and each segment was positioned lumen slide down onto the Matrigel. Outgrowth of cells from the tissue is observed after 7 days (A). These cells are in part CD106 + cells as observed after CD106 immuno-staining (Nuclei were counterstained with DAPI (Blue) (B). Cells were trypisinized and sorted after CD105-PE labelling using flow cytometry and after sorting 95% of cells are CD105 positive (C) with a typical endothelial cell morphology 5 days after plating (D). Long term culture were performed and immuno-histochemical labelling show that cells express CD31, CD106 CD105 and vWf (E). Characterization of ECs isolated from mice (F): List of transcripts detected in both Wt and PAI-1 −/− ECs by real time PCR. mRNA levels (fold induction) of ang2, CD106, CD54 and PAI-1 in Wt ECs 24 h after 10 Gy (G). Value 1 was attributed to un-irradiated Wt ECs. Results are mean of 3 independent experiments realized in triplicates. * p<0.05 versus un-irradiated Wt ECs.
(TIF)

Figure S5 **Effect of irradiation on PAI-1 mRNA level in HUVECs.** Irradiation increases rapidly PAI-1 mRNA level in HUVEC. Effect of irradiation on PAI-1 mRNA level 1, 4, 24 and 48 hours after 10 Gy. * p<0.05 versus unirradiated HUVEC cells. Results are mean +/− SEM of three independent experiments realized in triplicates.
(TIF)

Figure S6 **Effect of siPAI-1 on PAI-1 mRNA and protein level in HUVECs.** PAI-1 mRNA protein level in HUVEC 48 h after transfection in absence or presence of 100 nM siPAI-1 (A). Representative western blot (B) and quantification (C) of PAI-1 protein expression in HUVEC transfected for 48 h with or without 100 nM siPAI-1. Results are mean +/− SEM (n = 3) * p<0.05 versus HUVEC cells transfected with 100 nm of non-targeting siRNA.
(TIF)

Figure S7 **Human active PAI-1 recombinant has no effect on apoptosis-related genes profile.** mRNA levels of 93 genes involved in apoptosis were measured in HUVEC treated or not with a human active PAI-1 recombinant or a human Latent PAI-1 recombinant using a TaqMan Low Density apoptosis Array (TLDA) approach. Scatter plots analyses of Control versus irradiated cells (A) and irradiated versus irradiated and treated with human active PAI-1 recombinant are showed. Heat map analyses (C) and fold changes (D) versus control reveal that exogenous PAI-1 has no effect on apoptosis related-gene profile after irradiation.
(TIF)

Figure S8 **PAI-1 genetic deficiency is associated with increased Bcl-XL and Bcl-2 expression.** mRNA levels of Bcl-XL and Bcl-2 in Wt and PAI-1 −/− ECs 24 h hours after irradiation (A). Results are mean +/− SEM (n = 3). Representative western blots in Wt and Pai-1 −/− ECs 24 h hours after irradiation (B).
(TIF)

Table S1 **Bax, Bcl-2, Caspase 3, Survivin, Bcl-XL, Akt and PTEN mRNA levels in total intestinal tissues in Wt and PAI-1 −/− mice.** mRNA levels in total intestinal tissues in sham Wt and sham PAI-1 −/− mice 5 h (A) and 24 h (B) after surgery were determined by real time PCR. Results are +/− SEM (n = 6 mice/group).
(TIF)
