# Supplementary figures and images for: PAI-1-Dependent Endothelial Cell Death Determines Severity of Radiation-Induced Intestinal Injury
Source: PLoS One. 2012 Apr 26;7(4):e35740. doi: 10.1371/journal.pone.0035740 (PMC3338537; doi:10.1371/journal.pone.0035740)

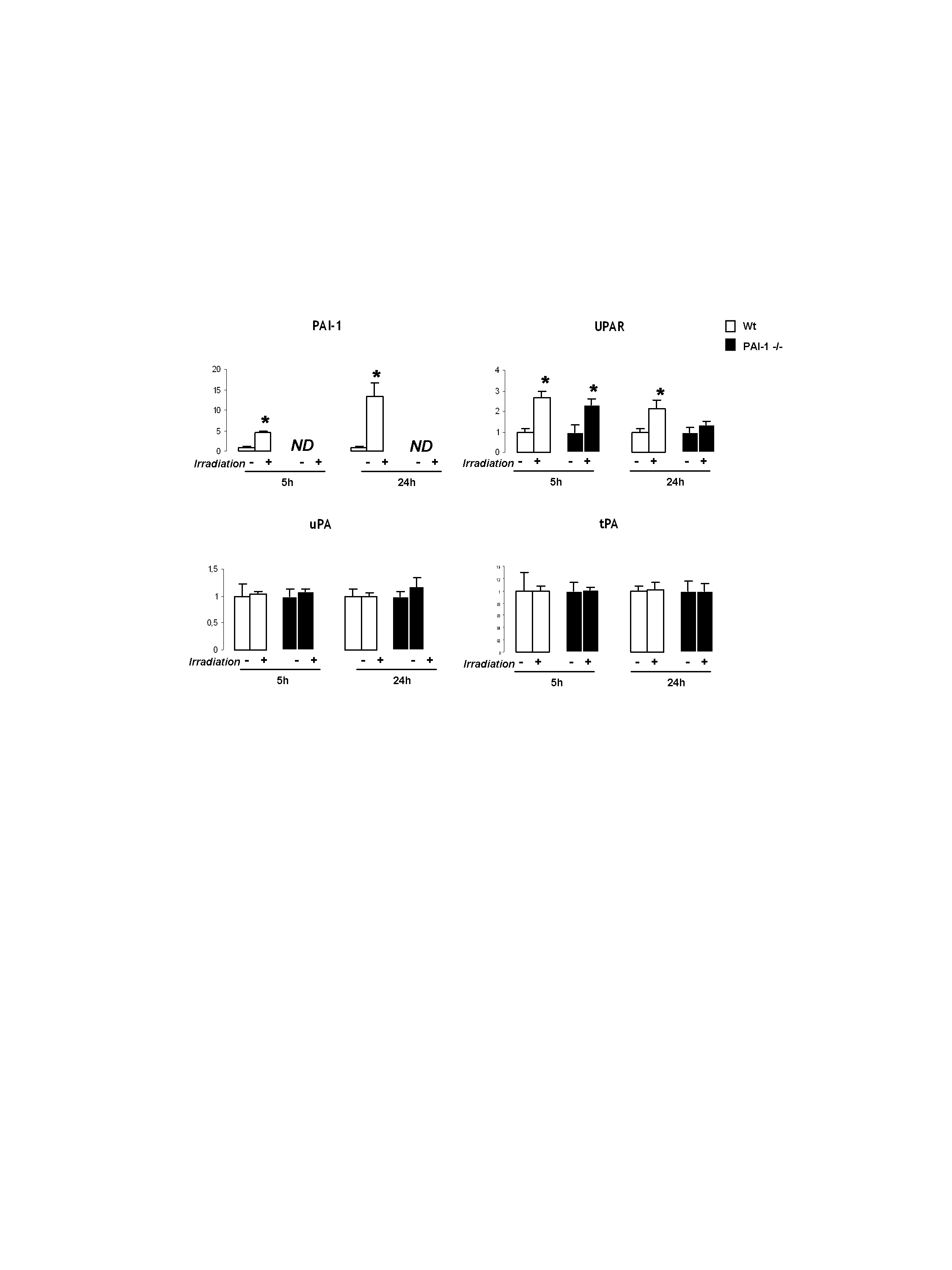

Supplement: Figure S1 — Effect of irradiation on mRNA levels of PAI-1, uPAR, uPA and tPA in Wt and PAI-1 −/− mice. mRNA levels of plasminogen activation system in Wt and PAI-1 −/− mice in total intestinal tissues were measured by Real time PCR 5 h and 24 h after irradiation. Results are +/− SEM (n = 6 mice/group). ND : not detected. (TIF) [file pone.0035740.s001.tif]

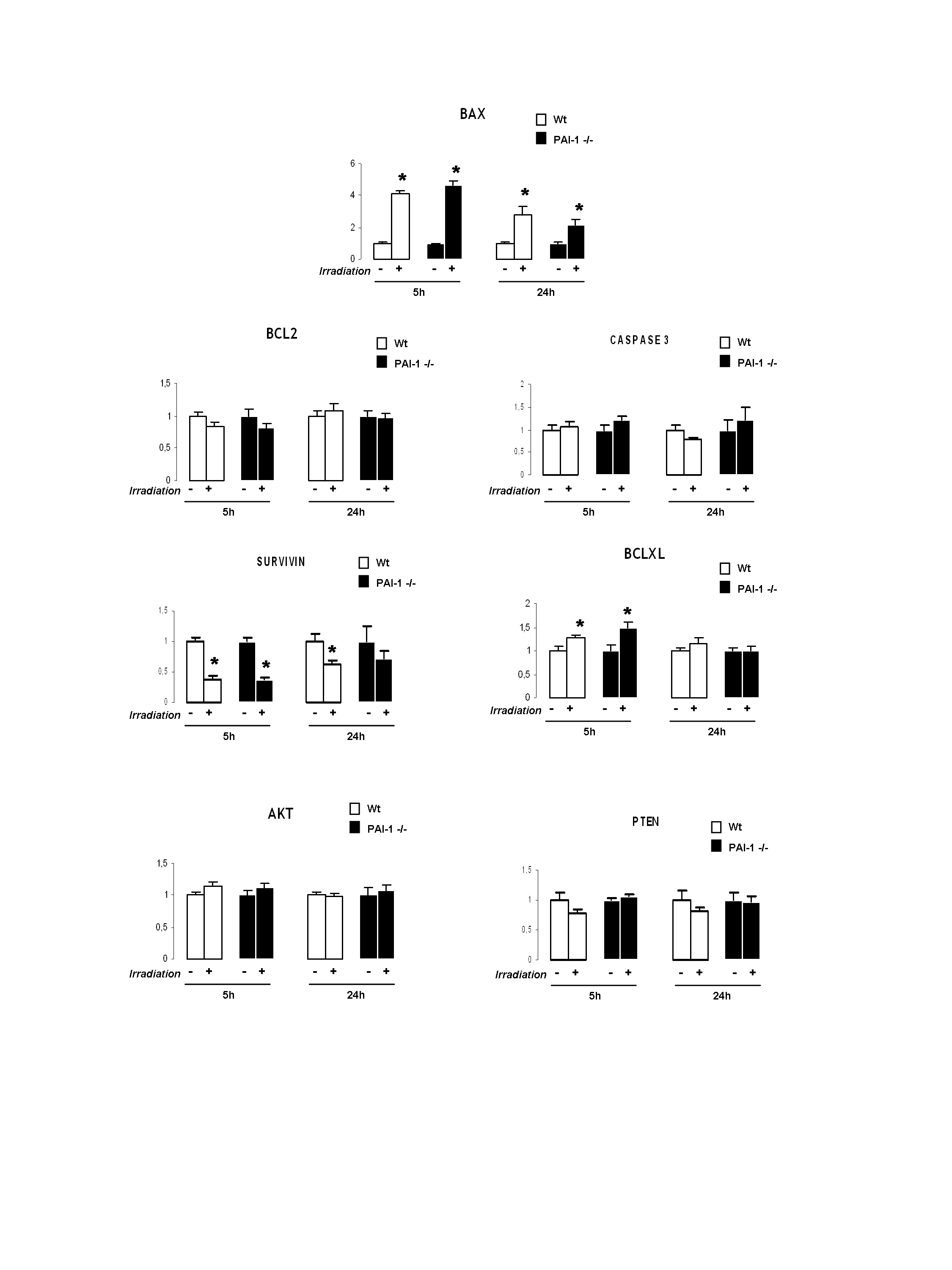

Supplement: Figure S2 — Effect of irradiation on mRNA levels of Bax, Bcl-2, Caspase 3, Survivin, Bcl-XL, Akt and PTEN in Wt and PAI-1 −/− mice. mRNA levels in Wt and PAI-1 −/− mice in total intestinal tissues were measured by Real time PCR 5 h and 24 h after irradiation. Results are +/− SEM (n = 6 mice/group). (TIF) [file pone.0035740.s002.tif]

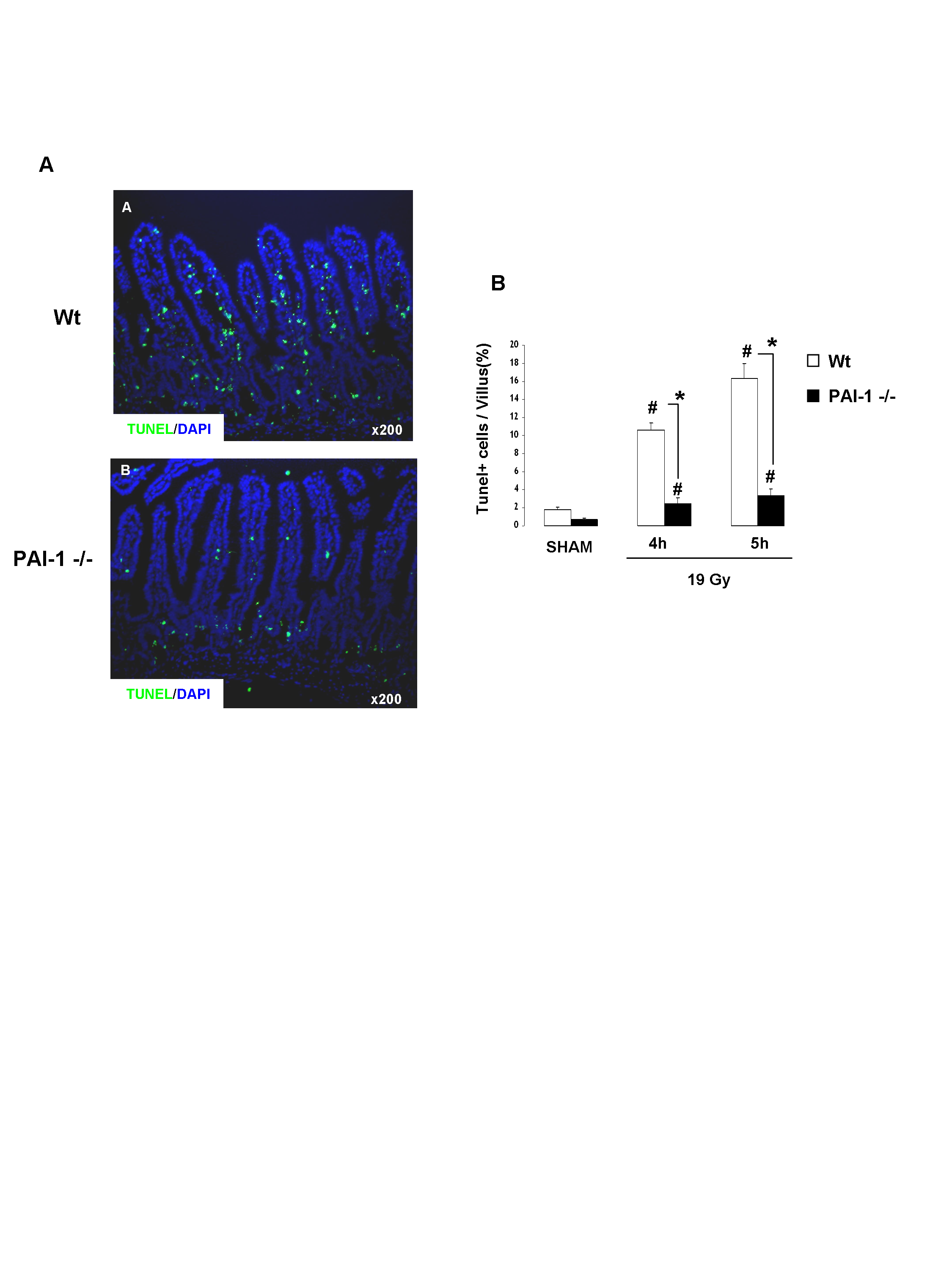

Supplement: Figure S3 — PAI-1 genetic deficiency is associated with reduced radiation-induced intestinal apoptosis. Three cm of intestine from Wt and PAI-1 −/− mice was irradiated with a localized 19 Gy single dose. Apoptosis in irradiated intestine was assessed by TUNEL staining. (A) Representative TUNEL staining (green) in Wt (A) and PAI-1 −/− (B) mice 4 hours after irradiation. Nuclei were counterstained with DAPI (blue). (B) Quantitative assessment of TUNEL+ cells in the villus in Wt and PAI-1 −/− mice 4 and 5 hours after irradiation. (n = 6 mice/group) # p<0.05 versus sham mice with the same genotype. # p<0.05 between irradiated Wt and PAI-1 −/− mice. (TIF) [file pone.0035740.s003.tif]

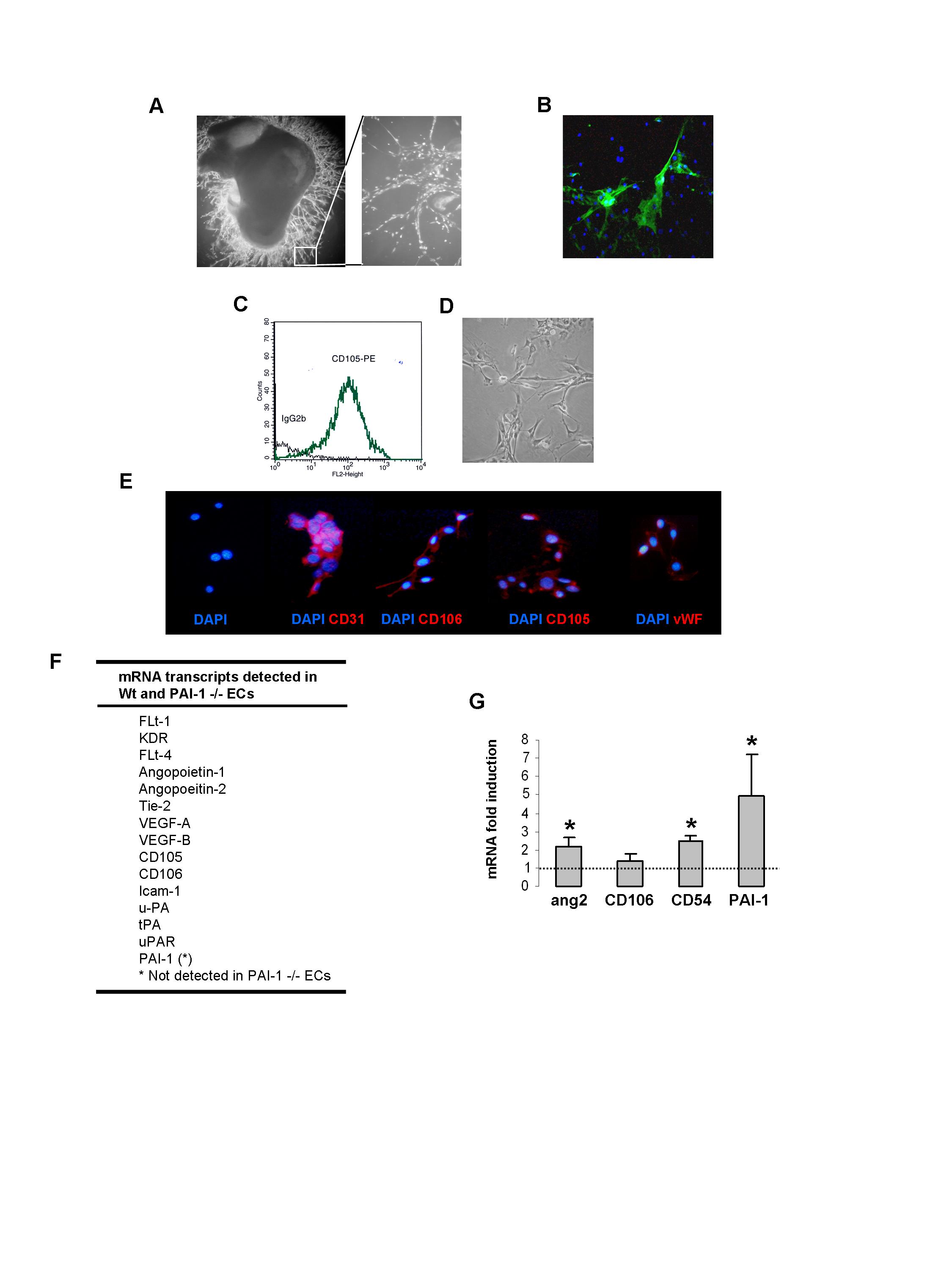

Supplement: Figure S4 — Preparation and characterization of mice endothelial cells isolated from aortas. The aorta was harvested cut into pieces, opened longitudinally, and each segment was positioned lumen slide down onto the Matrigel. Outgrowth of cells from the tissue is observed after 7 days (A). These cells are in part CD106 + cells as observed after CD106 immuno-staining (Nuclei were counterstained with DAPI (Blue) (B). Cells were trypisinized and sorted after CD105-PE labelling using flow cytometry and after sorting 95% of cells are CD105 positive (C) with a typical endothelial cell morphology 5 days after plating (D). Long term culture were performed and immuno-histochemical labelling show that cells express CD31, CD106 CD105 and vWf (E). Characterization of ECs isolated from mice (F): List of transcripts detected in both Wt and PAI-1 −/− ECs by real time PCR. mRNA levels (fold induction) of ang2, CD106, CD54 and PAI-1 in Wt ECs 24 h after 10 Gy (G). Value 1 was attributed to un-irradiated Wt ECs. Results are mean of 3 independent experiments realized in triplicates. * p<0.05 versus un-irradiated Wt ECs. (TIF) [file pone.0035740.s004.tif]

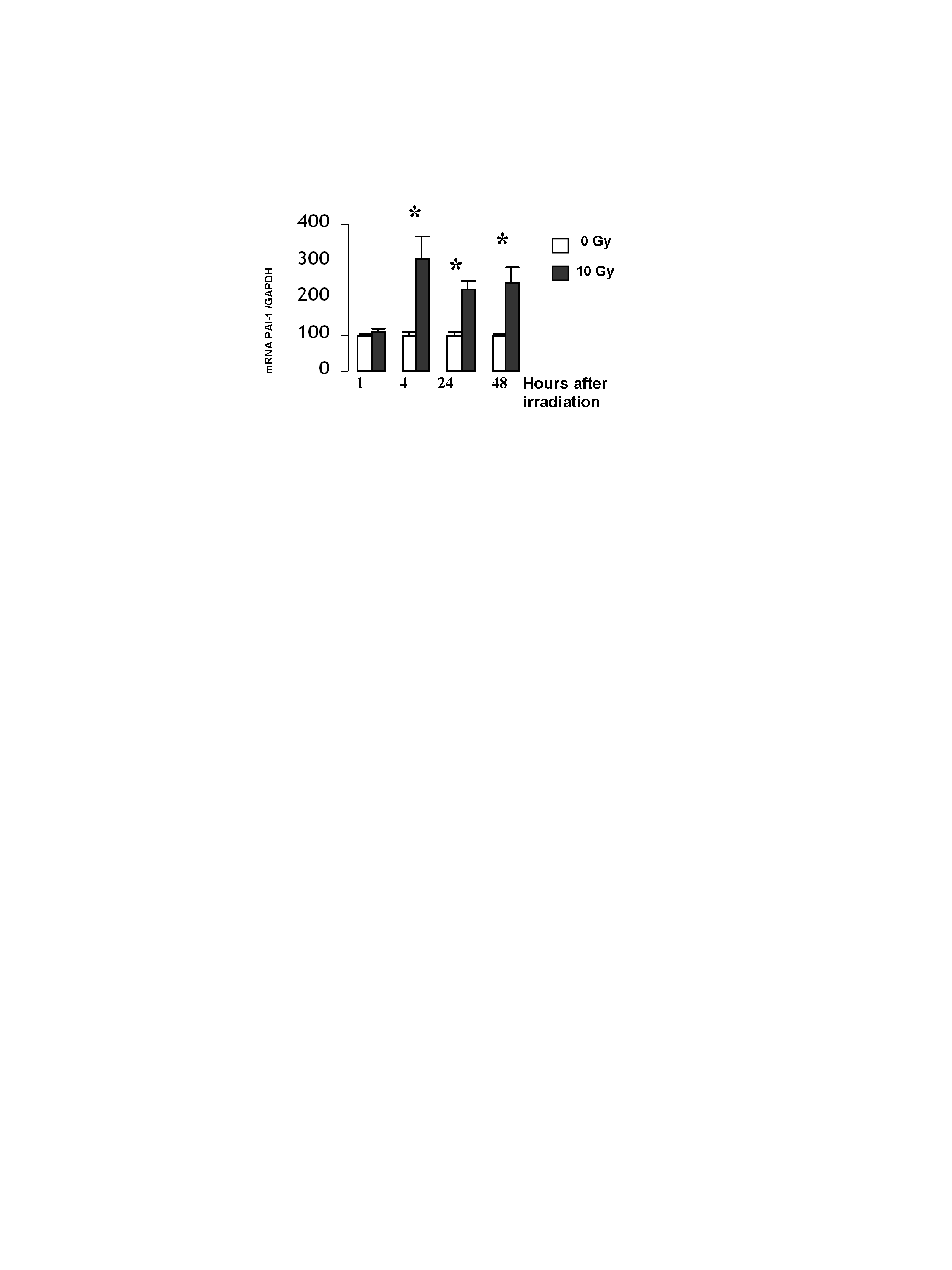

Supplement: Figure S5 — Effect of irradiation on PAI-1 mRNA level in HUVECs. Irradiation increases rapidly PAI-1 mRNA level in HUVEC. Effect of irradiation on PAI-1 mRNA level 1, 4, 24 and 48 hours after 10 Gy. * p<0.05 versus unirradiated HUVEC cells. Results are mean +/− SEM of three independent experiments realized in triplicates. (TIF) [file pone.0035740.s005.tif]

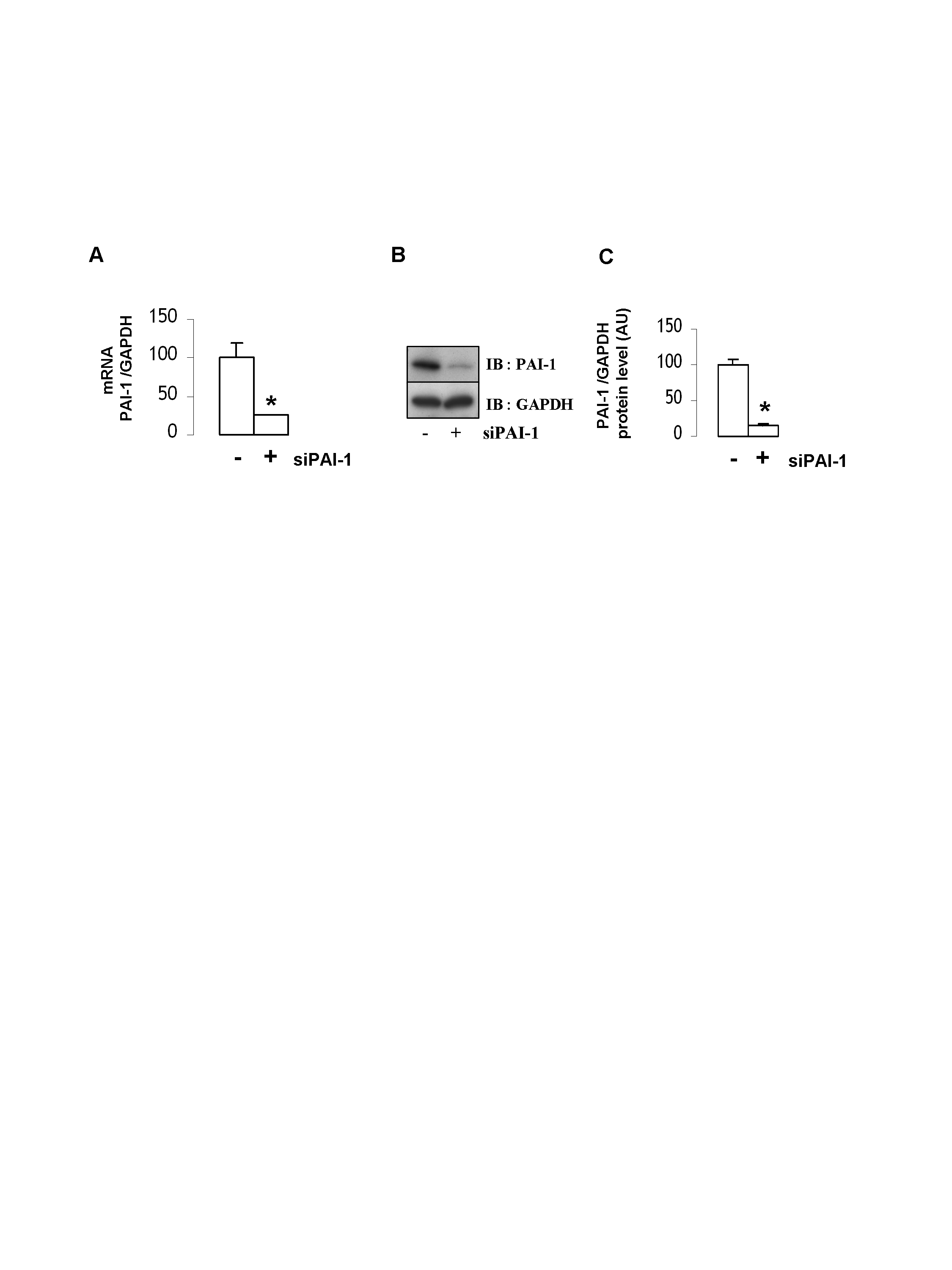

Supplement: Figure S6 — Effect of siPAI-1 on PAI-1 mRNA and protein level in HUVECs. PAI-1 mRNA protein level in HUVEC 48 h after transfection in absence or presence of 100 nM siPAI-1 (A). Representative western blot (B) and quantification (C) of PAI-1 protein expression in HUVEC transfected for 48 h with or without 100 nM siPAI-1. Results are mean +/− SEM (n = 3) * p<0.05 versus HUVEC cells transfected with 100 nm of non-targeting siRNA. (TIF) [file pone.0035740.s006.tif]

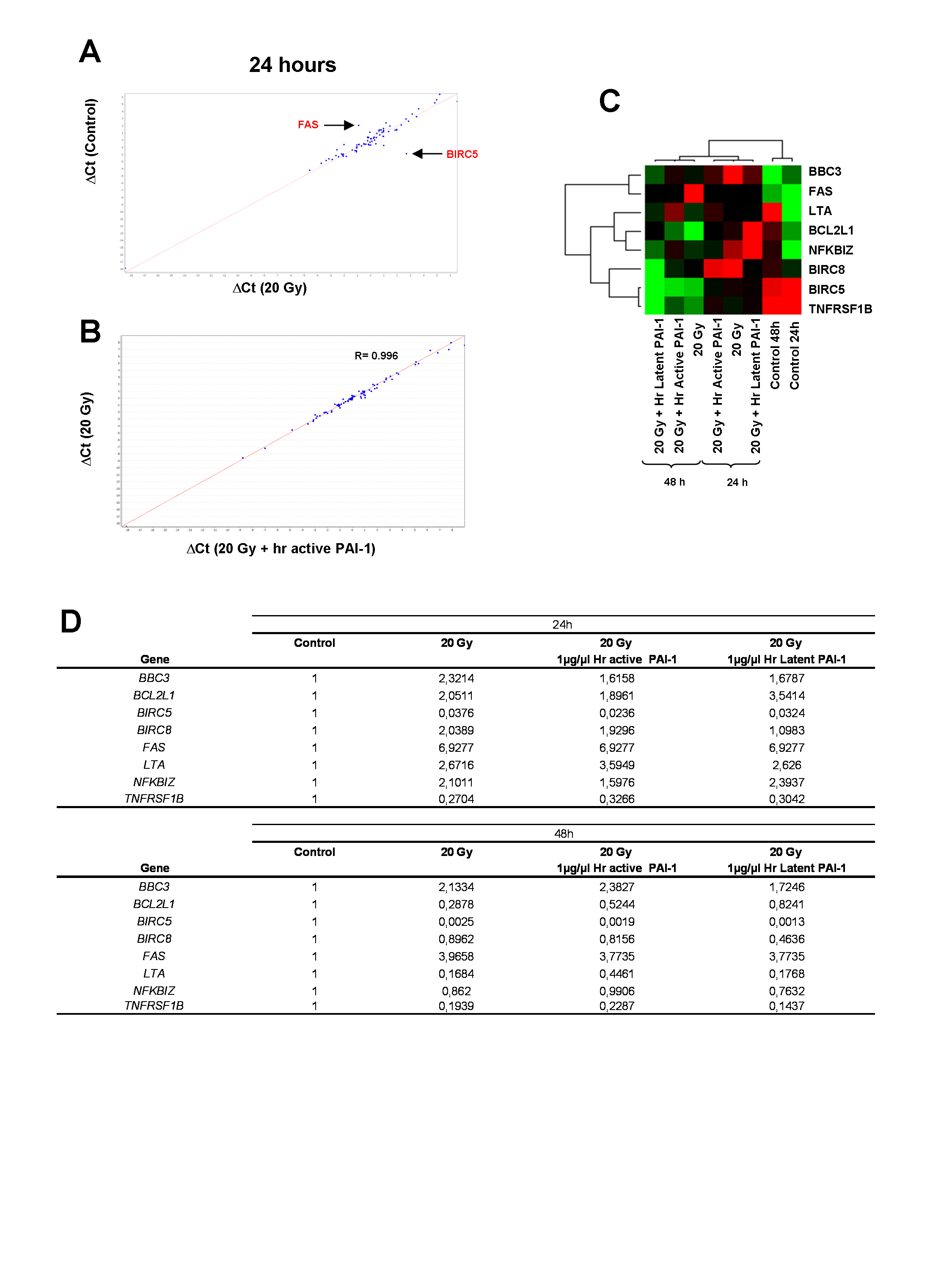

Supplement: Figure S7 — Human active PAI-1 recombinant has no effect on apoptosis-related genes profile. mRNA levels of 93 genes involved in apoptosis were measured in HUVEC treated or not with a human active PAI-1 recombinant or a human Latent PAI-1 recombinant using a TaqMan Low Density apoptosis Array (TLDA) approach. Scatter plots analyses of Control versus irradiated cells (A) and irradiated versus irradiated and treated with human active PAI-1 recombinant are showed. Heat map analyses (C) and fold changes (D) versus control reveal that exogenous PAI-1 has no effect on apoptosis related-gene profile after irradiation. (TIF) [file pone.0035740.s007.tif]

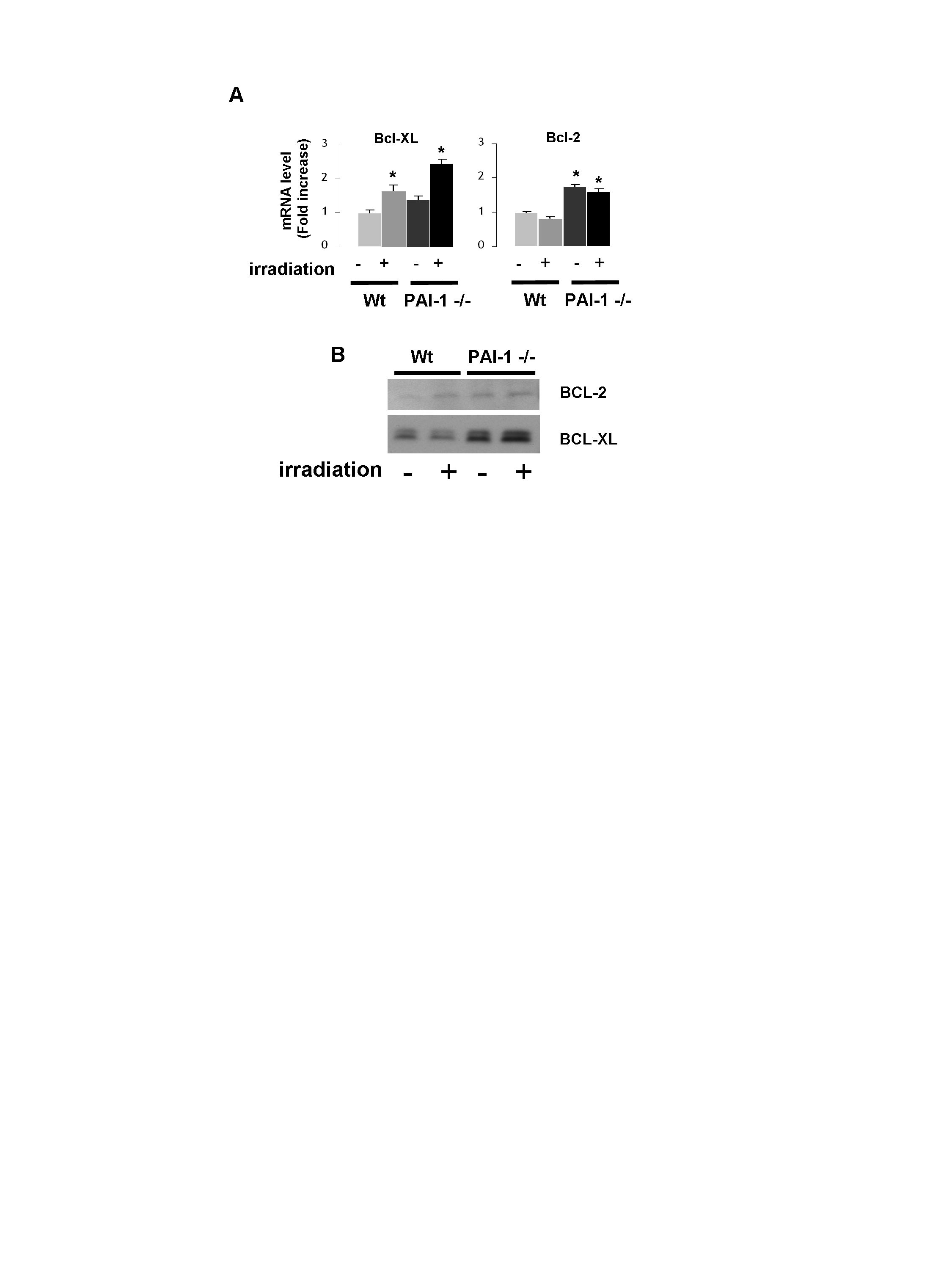

Supplement: Figure S8 — PAI-1 genetic deficiency is associated with increased Bcl-XL and Bcl-2 expression. mRNA levels of Bcl-XL and Bcl-2 in Wt and PAI-1 −/− ECs 24 h hours after irradiation (A). Results are mean +/− SEM (n = 3). Representative western blots in Wt and Pai-1 −/− ECs 24 h hours after irradiation (B). (TIF) [file pone.0035740.s008.tif]

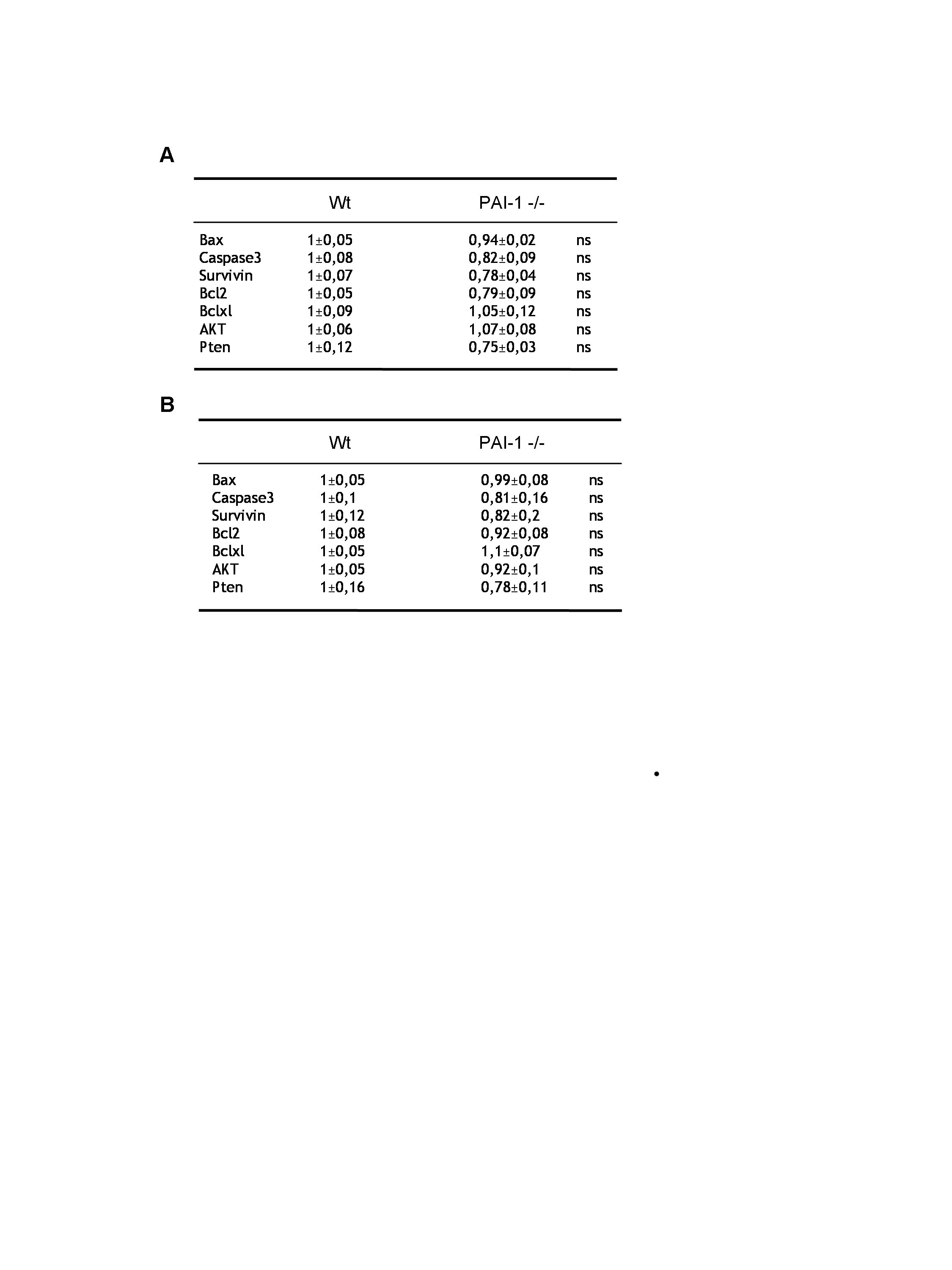

Supplement: Table S1 — Bax, Bcl-2, Caspase 3, Survivin, Bcl-XL, Akt and PTEN mRNA levels in total intestinal tissues in Wt and PAI-1 −/− mice. mRNA levels in total intestinal tissues in sham Wt and sham PAI-1 −/− mice 5 h (A) and 24 h (B) after surgery were determined by real time PCR. Results are +/− SEM (n = 6 mice/group). (TIF) [file pone.0035740.s009.tif]
